# Effectiveness of an environmental nutrition and physical activity intervention in early childhood education and care settings (NAPSACC UK): a multicentre cluster randomised controlled trial

**DOI:** 10.1016/j.lanepe.2025.101550

**Published:** 2025-12-18

**Authors:** Ruth Kipping, Sharon Anne Simpson, Kim Hannam, Peter S. Blair, Russell Jago, Corby K. Martin, Zoi Toumpakari, Laura Johnson, James Garbutt, Rachel Maishman, James White, Rebecca Langford, William Hollingworth, Madeleine Cochrane, Laurence Moore, Chris Metcalfe, Anne Martin, Stephanie Chambers, Thomas Reid, Megan Pardoe, Alexandra Dobell, Marie Murphy, Susan Stratton, Jemima Cooper, Jodi Taylor, Miranda Pallan

**Affiliations:** a Bristol Medical School, University of Bristol, Bristol, UK; b MRC/CSO Social & Public Health Sciences Unit, University of Glasgow, Glasgow, UK; c Pennington Biomedical Research Centre, Baton Rouge, USA; d School for Policy Studies, University of Bristol, Bristol, UK; e School of Medicine, Cardiff University, Cardiff, UK; f School of Social and Political Sciences, University of Glasgow, Glasgow, UK; g Department of Applied Health Sciences, University of Birmingham, Birmingham, UK

**Keywords:** Pre-school, Nursery, Children, Physical activity, Nutrition, Early childhood education and care

## Abstract

**Background:**

Early childhood education and care (ECEC) provision is widespread. NAPSACC UK is an intervention in ECECs designed to improve nutrition and physical activity policies, practice and provision through ECEC staff workshops, self-assessment and assistance over one year. It was adapted for the UK from the USA and we tested whether it reduced energy consumption and increased physical activity.

**Methods:**

Repeated cross-sectional, multicentre, two-arm, single-blind, parallel-group, cluster-randomised controlled trial including ECEC providers in the UK. The randomisation was conducted by a statistician who was blinded to ECEC provider identity, with allocation within each local authority area and by ECEC Index of Multiple Deprivation scores to minimise differences between arms. Participants were not blind to allocation. Co-primary outcomes after 12-months were child average total energy consumed per eating occasion in the ECEC (lunch or snack) and child accelerometer-assessed total physical activity on ECEC days. Secondary outcomes were moderate-to-vigorous physical activity, sedentary time, energy served and consumed at lunch and snacks, diet quality, and Body Mass Index z-score. The senior statistician and majority of co-investigators were blinded. Analysis was intention-to-treat. Trial registration is ISRCTN33134697 and is completed.

**Findings:**

Between 14 March 2022 and 25 March 2024 we enrolled 52 ECEC providers (25 intervention; 27 control) and 835 2-5 year-olds (401 intervention, 434 control). The co-primary outcomes were assessed 12 months after baseline with data provided by 382 children for nutrition and 244 children for physical activity. There was no evidence of a difference in the co-primary outcomes compared to control of average kcal per eating occasion in ECEC (adjusted geometric mean ratio 0.86 (95% CI 0.72–1.03; p = 0.09)) or total physical activity (adjusted mean difference (aMD) −2.13 min (95% CI −10.96 to 6.70; p = 0.64)). There was evidence of lower lunch energy served (aMD −69.1 kcal per occasion (95% CI −116 to −22.2; p = 0.004)) and consumed (aMD −67.7 kcal per occasion (95% CI −118.6 to −18.7, p = 0.009)) with the intervention. There was no evidence of differences in other secondary outcomes. No adverse events were reported.

**Interpretation:**

NAPSACC UK did not improve average kcal per eating occasion in ECEC or physical activity. Lower lunch energy servings and consumption closer to recommendations were observed as secondary outcomes. The lower fidelity to the intervention than intended and staffing pressures give insight into interpretation of the null result. Therefore, we recommend that policy-level and statutory changes, which require low agency by individual ECEC settings are research and policy priorities for nutrition and physical activity in ECEC.

**Funding:**

National Institute for Health and Care Research (NIHR):127551.

**Research in context**

**Evidence before this study**

Early childhood education and care (ECEC) provides a scalable setting for promoting health of children aged under five through policies and practices relating to nutrition and physical activity. We conducted a literature review using PubMed and Cochrane Library, without language restrictions, from 1980 to 2025. Our search strategy used the following terms: (“physical activity” OR exercise OR obesity OR “obesity prevention” OR “obesity intervention” OR nutrition OR diet) AND (preschool OR nursery OR childcare OR ECEC) to identify studies looking at physical activity and nutrition in early years settings. Previous research has identified that healthy eating interventions may have favourable effects on weight and risk of overweight and obesity in children, although the evidence is uncertain on the positive effect of ECEC-based healthy interventions on children's diet quality. Systematic reviews of obesity prevention, physical activity and nutrition in young children have identified a clear need for more research in this area with robust study designs and very few trials in the UK. There is a recognised gap in effective physical activity and nutrition interventions in ECEC for children aged 0–5 years. Search was carried out 13th–17th January 2025 with no language restrictions.

**Added value of this study**

NAPSACC is an environmental intervention developed in the US which aims to improve nutrition and physical activity policies, practice and provision in ECEC settings. We tested whether an adapted one-year NAPSACC intervention (with two cycles of nutrition and physical activity ECEC self-assessment, staff workshops, goal setting and support) reduced energy consumption and increased total physical activity in 2–5-year-old children in the UK. We found that children who were exposed to the NAPSACC UK intervention in ECEC settings did not have lower calorie intake averaged across eating occasion nor increased total physical activity during ECEC time. There was some evidence the intervention led to significant improvements in the secondary outcomes of energy served and consumed at lunch. We saw no evidence of change in any of the other measures of physical activity or change in measures of adiposity.

**Implications of all the available evidence**

Our trial findings do not support the roll-out of NAPSACC in the UK. The UK context of restricted public health funding and constrained ECEC sector capacity suggests that policy-level and statutory changes, which require lower agency, may be more fruitful endeavors. Mandated nutritional values and portion sizes, with free provision of lunches may provide greater potential for comprehensive reach and reduction in health inequalities. Our recommendation is that research and policy should focus on the provision, acceptability and value of policy and statutory changes for nutrition and physical activity in ECEC.

## Introduction

Early childhood is a priority period for dietary intake and physical activity to support child development and prevent a range of chronic conditions.^1^ Yet, internationally, young children do not meet dietary or physical activity recommendations^2^^,^^3^ and over 5% of children globally aged under 5 are overweight.^4^ In England and Scotland 22% of children in the first year of school (aged 4–6 years) were living with overweight or obesity in 2023/24. Three-quarters of three-year-olds in Organisation for Economic Cooperation (OECD) countries are enrolled in early childhood education and care (ECEC). ECEC provides a scalable setting for promoting child health.^5^ In England children aged 0–4 attend ECEC for an average of 22 h per week; 15 h of which are funded by the Government for 3–4 year olds with children of working parents eligible for up to 30 h per week. In England food is not funded by the Government and provision is from parents or providers; in Scotland children attending ECEC receive free lunches. Government policies are often insufficient for promoting nutrition and physical activity in ECEC settings: such as only requiring “outdoor activity once a day”^6^; not mandating nutritional standards for food served; and not providing free lunches.^7^

A review of ECEC healthy eating interventions found uncertainty on the impact on child diet quality, with little to no difference in child consumption of non-core foods^8^ (foods surplus to nutritional requirements, such as sugar-sweetened beverages) and little to no difference in measures of overweight or obesity.^5^ Most of the trials were conducted in the USA and Australia with only three conducted in the UK.^5^ Another review of strategies to promote healthy eating, physical activity and obesity prevention policies, practices, or programmes within childcare services found that while strategies probably improve policy, practice or programme implementation, there was no evidence for improvements in measures of child diet, physical activity or weight status and no trials in the UK.^8^

NAPSACC (Nutrition and Physical Activity Self-Assessment for Child Care) is an environmental intervention developed in the US which aims to improve nutrition and physical activity policies, practice and provision, with evidence demonstrating impacts on the ECEC environment and child outcomes.^9^ NAPSACC UK was adapted from the US and a feasibility trial has been conducted with the adaptations, duration, frequency and content detailed in prior publications.^10^^,^^11^ To assess effectiveness of the NAPSACC UK intervention, we conducted a multi-centre trial with embedded process and economic evaluations of the NAPSACC UK intervention, which will be separately reported.^12^ We aimed to test whether the adapted one-year NAPSACC UK intervention, with two cycles of nutrition and PA self-assessment, staff workshops, goal setting and support, reduced energy consumption and increased total physical activity in 2–5-year-old children in the UK.

## Methods

### Study design

NAPSACC UK was a repeated cross-sectional multi-centre, parallel-group, two-arm, cluster randomised controlled trial, incorporating process^12^ and economic evaluations.^9^ ECEC providers were randomised to receive either the NAPSACC UK intervention or to continue with usual practice for one year. The trial registration was ISRCTN33134697. The trial was approved by the Faculty of Health Sciences Research Ethics Committee at the University of Bristol, which included processes for reporting adverse events (REF: 6373) on 09/10/2019. The protocol^9^ and statistical analysis plan^13^ are publicly available. Initial recruitment started in November 2019–March 2020, was paused due to the COVID-19 pandemic and restarted in February 2022.

### Participants

ECEC providers were day nurseries, private or local government nursery schools, and nursery classes or pre-schools. These were recruited from four local authority areas with a broad range of deprivation status, ethnicity and urban and semi-rural locations in England and Scotland. Fifty-two ECEC providers were recruited (19 Somerset, 15 Ayrshire and Arran, nine Swindon, and nine Sandwell). ECEC providers were eligible if they had a minimum of 15 children aged 2–4 years attending for at least 12 h per week who ate their lunch at the ECEC provider; lunches could be provided by the ECEC or by parents. Children were eligible to participate if they were enrolled at the ECEC provider and were: at least 2 years old at the time of data collection; not yet attending primary school; attending for a minimum of 12 h a week across the year or 15 h a week during term time; and they had lunch at least once a week (ECEC or parent provided) to ensure that exposure to any changes in nutritional and activity polices had the potential for change.

Written informed consent for random allocation was obtained from a member of each ECEC senior leadership team following an eligibility check and prior to baseline data collection and randomisation. Parents of all eligible children within a consented ECEC setting had the opportunity to review study information documents as hard copies or online and view a short online video. Opt-in written consent was obtained for parents as participants, as well as on behalf of their child(ren). After baseline data collection ECEC providers were randomised to receive the NAPSACC UK intervention for 12 months with a staggered start from September 2022 or continue with their usual practice. ECEC providers were given two payments of £300 as a thank you for taking part and parents were given a payment of £10 for completed data collection at baseline and £20 at follow-up.

We anticipated some children would leave the ECEC when they reached the age for registering at schools or would change ECEC provider. As the environmental nature of the intervention aimed to expose all children to changes in intervention practices, additional children were recruited from participating ECEC providers towards the end of the 12-month intervention period, prior to follow-up data collection in a repeated cross-sectional trial design.

### NAPSACC UK intervention

Fifteen local authority or health board public health specialists were trained as ‘NAPSACC UK Partners’ during a two-day training session delivered by nutrition and physical activity specialists and the Trial Manager (Table 1). The Partners were trained how to: a) deliver two workshops to ECEC practitioners on nutrition and physical activity; b) support provider completion of the ‘review and reflect’ self-assessment process; and c) provide ongoing assistance. NAPSACC UK Partners received a comprehensive manual outlining the content and structure for delivering the intervention.

**Table 1:** TiDIER description of NAPSACC UK.

Item	Description
Name	Nutrition and Physical Activity Self-Assessment for Child Care UK (NAPSACC UK)
Why	NAPSACC UK is an intervention delivered in child care settings with the aim of improving the nutrition and physical activity environment, through a process of self-assessment and targeted assistance. NAPSACC UK is a theory-based program that employs components of social cognitive theory (SCT) and the socio-ecological framework. The objectives of the programme are to improve the nutritional quality, variety and quantity of food served, amount and quality of physical activity, staff–child interactions and staff behaviours around nutrition and physical activity and child care provider policies.
What: materials	The NAPSACC UK intervention is based around a self-assessment tool completed by ECEC managers with advice and support from a NAPSACC UK “Partner”. This document, called the ‘Review & Reflect’, is an 101-item multiple choice questionnaire, completed by the ECEC manager, covering areas in nutrition, physical activity and play, outdoor play and learning, and screen time. Following completion of the Review & Reflect, the ECEC manager along with the NAPSACC UK Partner agree on eight goals; three nutrition, three physical activity and a further two of the setting's choice.
What: procedures	The NAPSACC UK intervention is a five stage process: 1 Self-Assessment. 2 Workshop delivery: Specialised staff deliver workshops to all ECEC staff on: i) Nutrition; ii) Physical Activity. 3 Goal setting and Action Planning: The NAPSACC UK Partner works with the ECEC manager to develop an action plan, listing eight goals for improvement. 4 Tailored technical assistance: NAPSACC UK Partner continues regular contact with ECEC to provide support and advice toward them meeting their goals. 5 Evaluate, revise, repeat. The Review & Reflect self-assessment is repeated by the ECEC manager after six months and reviewed with the NAPSACC UK Partner to see where improvements have been made or not, and to explore ways to overcome barriers; action plans are revised to set eight new goals for the next six months.
Who provided	NAPSACC UK Partners and Local Authority/Health Board staff who deliver the ECEC workshops are chosen locally from a range of health or health improvement staff with appropriate skills. All staff are provided with one day of training led by specialists in nutrition and physical activity who provided the training in the feasibility study. The partners deliver the intervention in addition to their Local Authority role.
How	The main part of the intervention is delivered face to face; this includes Partners going through the Review & Reflect, action planning and attending or delivering the workshops (depending on whether the Partners are also the staff delivering the workshops). Other parts of the intervention, such as on-going support and advice from the NAPSACC UK Partner is provided over the phone, by email or face to face. All parts of the intervention are delivered to participating ECEC settings individually. Some parts may be delivered on a one-to-one basis (e.g. ECEC manager and NAPSACC UK Partner setting goals), while other parts such as the workshops are delivered to a group of staff from one ECEC. Partners have four days contact with each ECEC over the 12 months.
Where	The NAPSACC UK intervention is delivered in the ECEC itself. The NAPSACC UK Partner offers visits to the ECEC and the workshops take place at the ECEC or an online recording.
When and how much	The NAPSACC UK intervention takes place over 12 months. The length of the workshops are a total of 6 h where they are delivered in person, followed by an online refresher workshop after 6 months; recorded workshops (without group interaction) are available where individual staff need flexibility to engage with the workshops.The ECEC settings receive ongoing regular support over the 12 months.
Tailoring	The technical assistance offered by the NAPSACC UK Partner depends on the goals.
Modifications	In the feasibility study the intervention was five months; in the full trial it is 12 months. NAPSACC was designed in the US to be for a year and this longer period enables a mid-intervention review of progress against goals and further goals to be sets. In the feasibility study the Partners were Health Visitors; in the full trial Local Authorities choose appropriate health staff.

NAPSACC UK Partners supported intervention ECEC settings to complete two six-month cycles of ‘review and reflect’ to review their physical activity and nutrition policies and practices against best practice standards. They delivered training to ECEC staff through group workshops, supported ECEC staff to set goals, and provided assistance when required. After the first cycle, the ‘review and reflect’ process was repeated setting additional goals. Although parents were not directly involved in the intervention (unlike in the feasibility study,^14^ there was no specific home component, however we added a lunchbox section of best practices to the review and reflect which included parental engagement in food provided from home). However, the ECECs may have made changes which had an element of parental involvement such as through education or policies. Further details of the intervention are provided in the published protocol, Table 1, and Appendix p2 and p3. Delivery of the intervention was staggered between September 2022 and February 2024 because of ECEC recruitment and Partner availability.

### Randomisation and masking

Once an ECEC confirmed its participation and baseline data had been collected, ECECs were randomised in a 1:1 ratio by a statistician from the Bristol Trials Centre, who was blinded to ECEC provider identity. Allocation of each ECEC provider was conducted within each local authority area and minimised by average English or Scottish Index of Multiple Deprivation (IMD) scores^15^^,^^16^ (created for each ECEC provider using the postcodes of the children recruited at baseline) to minimise baseline differences between arms. The senior statistician and co-investigators (except MP, SS and BL) were blind to allocation, and the study statistician was blind to allocation until the statistical analysis plan had been signed off; it was not possible to blind the intervention team, research staff collecting data, or process evaluation team.

### Outcomes

The co-primary outcomes were mean accelerometer-assessed total physical activity (TPA) on days the child attended the ECEC setting and energy (kcal) consumed per eating occasion averaged across snack and lunch eating occasions within ECEC settings. The selection of secondary outcomes was informed by our theory of change (Appendix p3).^11^ Secondary physical activity outcomes were: mean moderate-to-vigorous physical activity (MVPA) on ECEC provider days; mean sedentary time on ECEC provider days; and the difference in mean TPA between days that the child attended the ECEC provider and weekdays they did not attend. Secondary nutrition outcomes were: energy (kcal/occasion) served at lunch time; energy (kcal/occasion) served at snack-time; energy (kcal/occasion) consumed at lunch time; energy (kcal/occasion) consumed at snack-time; and as an indicator of diet quality, percentage of total consumed lunch energy and the percentage of total consumed snack energy (kcal) from non-core food (%) (foods surplus to nutritional requirements,^17^ such as fruit-flavour low-calorie drinks, salted butter and bread sticks). Other secondary outcomes were child zBMI and the proportion of children living with overweight/obesity (using UK90 zBMI reference curves^18^). Outcomes were measured after a median of 12 months (IQR 11.8, 12.9 months), with the average value at baseline within each ECEC provider used for adjustment in statistical models. A process evaluation assessed the fidelity, acceptability and sustainability of the intervention.^12^

### Sample size

The study aimed to recruit 56 ECEC providers (784 children) assuming an average of 14 children recruited at each ECEC provider, allowing for two provider withdrawals and up to 35% of children failing to produce valid accelerometer data on days attending the ECEC provider. This would allow detection of a difference of 17 min TPA (based on results from the feasibility trial^10^) with 90% power at the 5% significance level, assuming an intra-cluster correlation of 0.087^19^ and coefficient of variation in cluster size of 0.3.^20^ This sample size would also allow for the detection of 0.4 standard deviations difference on the nutrition outcome under the same assumptions (assumed to be 45 kcal based on feasibility data^9^). Evidence of an impact on both primary outcome measures is required to support the adoption of NAPSACC UK into routine practice. In this situation, the two co-primary outcomes do not increase the probability of a false-positive conclusion, and no adjustment of the significance level was required. Full details on the sample size calculation are given in the statistical analysis plan.^13^

### Data collection

Baseline data refers to data collected on children enrolled at T0, and follow-up data refers to data collected on children at T1; due to the repeated cross-sectional design, the cohorts of children were different between the two time points with a small proportion of children included in both cohorts. Demographic data (including ethnicity, sex, date of birth and postcode to derive IMD) and usual child attendance were collected via an electronic or paper-based questionnaire completed by parents/carers at the point of recruitment (at T0 for children recruited at the start of the study, and before T1 data collection for those enrolled at T1). Anthropometric measurements were collected by trained field workers in a quiet area within the ECEC setting with a member of ECEC staff present.^9^ The Remote Food Photography Method (RFPM) was used to provide a direct estimation of food item energy and portion size.^21^^,^^22^ Food composition to give energy was based on the UK National Diet and Nutrition Survey nutrient databank, downloaded from the UK data archive.^23^ Accelerometers (ActiGraph GT3X+) were issued in the ECEC setting by research staff and parents were asked to ensure their child wore them for five consecutive weekdays while awake. Physical activity data were downloaded using the ActiLife software. Mediator questionnaires, used to measure knowledge and motivation around physical activity and nutrition in addition to how able respondents feel to provide these, were given to ECEC staff and parents/carers for completion either online or on paper. Process evaluation data as detailed in the protocol included: observations of Partner and ECEC staff training workshops, ECEC staff questionnaires, and interviews/focus groups with Partners, ECEC managers, commissioners and the research team.

### Statistical analyses

Analyses were carried out according to a pre-specified statistical analysis plan with post-hoc analyses, conducted to gain further insight into the effect of the intervention, clearly indicated as such. Analyses of primary and secondary outcome measures were conducted according to the intention-to-treat principle; all children providing data were included in the analysis according to the allocation of their ECEC and missing data were not imputed. Details of the derivation of outcome variables are given in Appendix p4. The primary analyses were carried out using physical activity data recorded between 09:00 and 15:00 h.^24^ Sensitivity analyses were carried out using mean minutes of activity for each activity type including all data recorded over the day (06:00–00:00 h).

Multilevel linear regression models were fitted with adjustment at the ECEC level (corresponding baseline outcome and local authority area as fixed effects, ECEC provider as a random effect to account for clustering) and at the child level (child deprivation status categorised into high [deciles 1–3], moderate [deciles 4–7] or low [deciles 8–10] deprivation as fixed effect). Due to the repeated cross-sectional design, baseline values for each of the outcomes were calculated as the average value observed across children within the ECEC provider at baseline. For the analysis of physical activity outcomes, as specified in the statistical analysis plan, the mean wear time for each child was fitted as a continuous covariate to adjust for the varying length of wear time across children. For the analysis of the nutritional co-primary outcome of kcal consumed per eating occasion, averaged across snack and lunch eating occasions, the type of eating occasion (lunch, morning snack, or afternoon snack) was fitted as a categorical covariate in the multilevel models. A log transformation was used to account for the systematic differences in size of lunch and snacks and effect estimates were reported as the geometric mean ratio (GMR). Multilevel logistic regression models were fitted for binary outcome models with adjustment at the ECEC level (local authority area as fixed effect, ECEC provider as random effect) and at the child level (child deprivation status). Sensitivity analyses, including adjustment for additional covariates with imbalance between groups at baseline, were performed for the co-primary outcomes (Appendix p5). A two-sided 5% significance level was used for all analyses, and likelihood ratio tests were performed for all effect estimates.

We assessed differential effects of the intervention on the co-primary outcomes according to pre-specified subgroups (local authority area, age, sex, child deprivation status and ECEC setting size) through the inclusion of the interaction term between trial arm and subgroup variable. A post-hoc subgroup analysis was performed for the primary nutrition outcome by country due to state funded food provision in Scotland but not in England. Further post-hoc analyses to explore the effect of NAP SACC UK on nutritional outcomes by food provision (ECEC provided vs parent provided lunchboxes) were also performed. Analyses were performed using Stata software, version 18.5 (StataCorp). This trial was overseen by an independent Trial Steering Committee.

### Role of the funding source

The funder (NIHR) approved the study design but had no role in original study design, data collection and analysis, data interpretation manuscript preparation, or the decision to submit for publication.

## Results

**Fig. 1:**
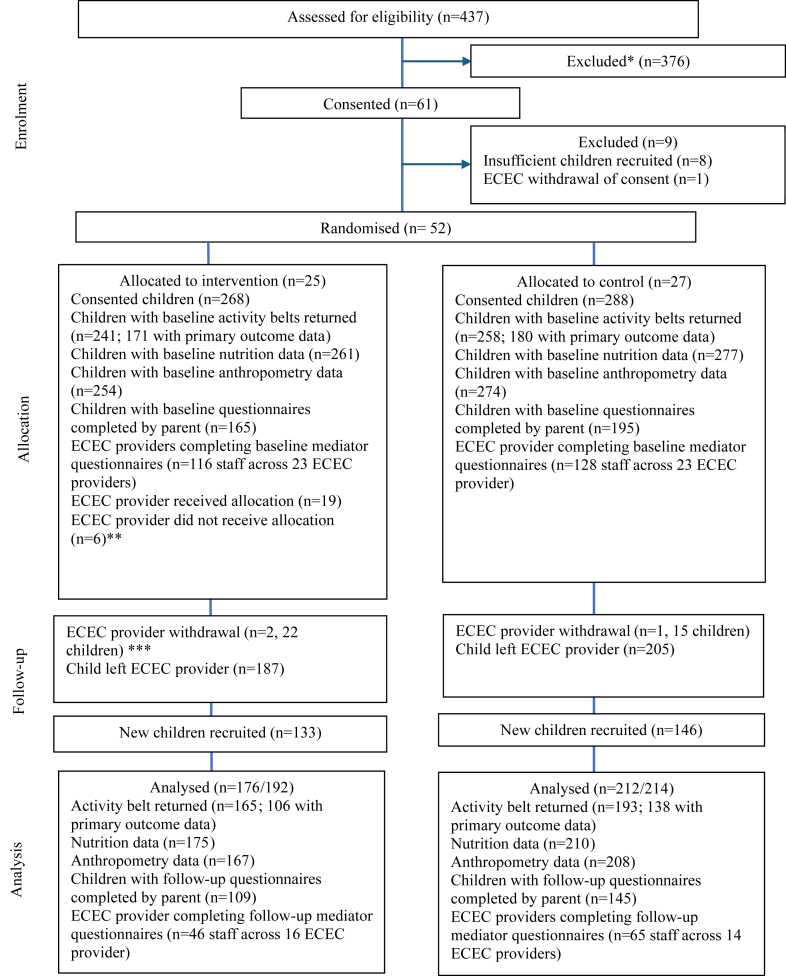
**Trial progression**. Abbreviations: ECEC, early childhood education and care. ∗Reasons include ECEC provider ineligible or did not respond to initial invitation. ∗∗Six ECEC providers did not engage with the NAP SACC UK intervention due to staffing pressures. Two of these ECEC providers formally withdrew from follow-up data collection. ∗∗∗ECEC provider withdrawal because the provider closed. Children analysed included children who provided outcome data for at least one of the included outcomes and were either recruited at T0 and did not leave the study during the follow-up period, or were recruited at T1.

Fig. 1 shows the trial profile. Fifty-two ECEC providers were randomised in the study (835 children). Twenty-five ECEC providers were allocated to the NAP SACC UK intervention (401 children) and 27 ECEC providers to the control arm (434 children) (Fig. 1). Three ECEC providers withdrew after randomisation (two in the intervention arm and one in the control arm). The COVID-19 pandemic, Brexit, and other contextual factors impacted ECEC staffing which impacted the recruitment and retention of ECEC providers (detailed in Appendix p6). Data at both T0 and T1 were available for 121 children; as expected most children (687) provided data for only one timepoint because of moves to schools or other ECEC settings. Baseline data (T0) were collected between 12th May 2022 and 6th February 2023; follow-up data (T1) were collected between 12th October 2023 and 22nd April 2024. No adverse events were reported.

Baseline ECEC provider characteristics were balanced between the two arms (see Appendix p7). Half of the ECEC providers had between 30 and 60 eligible children enrolled (range 16–144). Most ECEC providers were not attached to a school (31/52, 60%), were in England (37/52, 71%) and were categorised as being in areas of moderate deprivation (34/52, 65%). Due to the repeated cross-sectional trial design, child demographics were compared separately between the intervention and control arm at baseline and follow-up, and between baseline and follow-up within each treatment arm (Table 2). There were fewer children in intervention ECEC providers than control ECEC providers at baseline and follow-up. At baseline, more children in the control arm attended ECEC providers in Swindon, fewer attended ECEC providers in Ayrshire and Arran, and there were fewer 3-4 year-olds. At baseline children in intervention ECEC attended for a mean of 25.5 h (SD 7.9 h) per week, with a mean of 24.1 h (SD 8.1 h) per week in control settings. All other child demographic characteristics were balanced between arms at baseline. At follow-up, the intervention arm had fewer children from low deprivation areas compared to the control arm, fewer children from Swindon, more children from Sandwell, and more children under 3 years old. All other demographic characteristics were balanced between the arms at follow-up. When comparing follow-up with baseline, there were more children from high or moderately deprived areas and from Somerset in the intervention arm. In the control arm, fewer children were from areas of high deprivation, fewer were from Sandwell, and more were aged ≥3 years at follow-up compared to baseline.

**Table 2:** Baseline characteristics of trial participants.

	Demographics of children at T0				Demographics of children at T1			
	Intervention N = 261		Control N = 280		Intervention N = 176		Control N = 212	
	n/N or mean	% or SD	n/N or mean	% or SD	n/N or mean	% or SD	n/N or mean	% or SD
Male	118/259	45.6%	149/279	53.4%	80/175	45.7%	107/212	50.5%
Female	141/259	54.4%	130/279	46.6%	95/175	54.3%	105/212	49.5%
IMD category								
High deprivation	78/250	31.2%	106/269	39.4%	50/169	29.6%	53/203	26.1%
Moderate deprivation	119/250	47.6%	110/269	40.9%	98/169	58.0%	97/203	47.8%
Low deprivation	53/250	21.2%	53/269	19.7%	21/169	12.4%	53/203	26.1%
Local authority area								
Somerset	108/261	41.4%	116/280	41.4%	88/176	50.0%	95/212	44.8%
Swindon	19/261	7.3%	43/280	15.4%	10/176	5.7%	34/212	16.0%
Sandwell	42/261	16.1%	40/280	14.3%	28/176	15.9%	14/212	6.6%
Ayrshire and Arran	92/261	35.2%	81/280	28.9%	50/176	28.4%	69/212	32.6%
Age (mean, SD)	43.2	7.8	43.1	8.2	45.0	8.4	46.6	7.1
24–35 months	42/261	16.1%	62/279	22.2%	28/174	16.1%	18/212	8.5%
36–47 months	143/261	54.8%	126/279	45.2%	84/174	48.3%	104/212	49.1%
≥48 months	76/261	29.1%	91/279	32.6%	62/174	35.6%	86/212	42.5%
Ethnicity^a^								
White	225/259	86.9%	241/278	86.7%	147/175	84.0%	193/211	91.5%
Black	7/259	2.7%	1/278	0.4%	3/175	1.7%	1/211	0.5%
Asian	11/259	4.2%	17/278	6.1%	9/175	5.1%	8/211	3.8%
Other	16/259	6.2%	19/278	6.8%	16/175	9.1%	9/211	4.3%
Number of hours attending ECEC per week (mean, SD)	25.5	7.9	24.1	8.1	24.3	9.0	24.3	7.2

Abbreviations: IMD, index of multiple deprivation; SD, standard deviation; ECEC, early childhood education and care. Missing data (intervention group, control group): age at T1 n = 2 (2, 0); number of hours attending ECEC per week at T0 n = 3 (1, 2). Sex was reported by parents at study enrolment.

a Ethnicity definitions based upon parental self-report of child's ethnicity: White (White British/White other); Black (Black British/Black Caribbean/Black African); Asian (Asian British/Indian/Pakistani); Other (Mixed/Other/Would prefer not to say).

At the 12-month follow-up there was weak evidence that Kcal consumed at lunch and snacks were lower in the intervention group; adjusted geometric mean ratio (aGMR) 0.86 (95% CI 0.72, 1.03), p = 0.094. There was no evidence of a difference in minutes of TPA; adjusted mean difference (aMD) −2.13 min (95% CI −10.96, 6.70), p = 0.64 (Table 3).

**Table 3:** Primary and secondary outcomes.

	Data period	Intervention ECEC providers			Control ECEC providers			Adjusted Mean Difference (95% CI)^a^	p-value
		N	Mean or median	SD or IQR	N	Mean or median	SD or IQR		
**Co-primary outcome**									
Total energy (kcal) consumed averaged across eating occasions (lunch and snacks)	*Data given under secondary outcomes below; average kcal consumption per child was not calculated, however analysis models included all eating occasions to estimate the difference in kcal consumed averaged across all eating occasions that the child was present for in the ECEC provider.*							Adjusted GMR 0.86 (0.72, 1.03)^b^	0.09
Minutes of Total Physical Activity^c^	T0	171	87.3	24.1	180	87.9	27.5	−2.13 (−10.96, 6.70)^d^	0.64
	T1	106	95.0	29.0	138	96.4	28.8		
**Secondary outcomes**									
Kcal Energy consumed^e^									
Lunch	T0	261	319.3	(212.1, 439.8)	271	319.6	(215.5, 439.0)	−67.7 (−118.6, −16.7)	0.009
	T1	172	341.8	(209.0, 441.5)	210	368.9	(246.5, 514.7)		
Morning snack	T0	252	71.3	(33.4, 128.2)	251	65.2	(30.4, 127.8)	−5.9 (−31.3, 19.6)	0.65
	T1	166	61.0	(30.7, 116.0)	174	77.0	(37.0, 131.0)		
Afternoon snack	T0	126	82.7	(43.8, 180.7)	119	95.0	(24.0, 152.8)		
	T1	80	74.7	(49.8, 131.8)	78	88.4	(37.0, 130.5)		
Kcal Energy served^e^									
Lunch	T0	261	426.8	(345.0, 564.1)	271	449.3	(325.6, 564.1)	−69.1 (−116.0, −22.2)	0.004
	T1	172	425.3	(307.1, 557.2)	210	471.4	(342.7, 641.4)		
Morning snack	T0	252	94.8	(51.0, 162.7)	251	77.6	(49.8, 142.3)	−1.9 (−38.7, 34.8)	0.92
	T1	166	83.8	(51.0, 126.9)	174	89.6	(48.4, 159.5)		
Afternoon snack	T0	126	114.6	(57.2, 221.8)	119	108.0	(46.1, 174.8)		
	T1	80	86.4	(59.1, 157.3)	78	99.8	(49.2, 151.3)		
Percent of food non-core^e^									
Lunch	T0	261	34.1	(8.9, 56.8)	268	42.2	(7.9, 65.3)	−5.35 (−14.74, 4.04)	0.26
	T1	171	31.4	(0.0, 54.7)	208	41.3	(17.3, 59.3)		
Morning snack	T0	239	0.0	(0.0, 31.6)	236	0.0	(0.0, 34.2)	−3.45 (−12.56, 5.67)	0.46
	T1	163	0.0	(0.0, 0.0)	170	0.0	(0.0, 28.1)		
Afternoon snack	T0	120	39.8	(0.0, 78.6)	107	4.3	(0.0, 49.8)		
	T1	77	0.0	(0.0, 49.9)	76	0.0	(0.0, 43.8)		

	Data period	Intervention ECEC providers			Control ECEC providers			Adjusted mean difference (95% CI)	p-value
		N	Mean or median	SD or IQR	N	Mean or median	SD or IQR		
**Secondary outcomes**									
Minutes of MVPA^c^	T0	171	12.3	(7.6, 16.8)	180	13.1	(8.1, 18.4)	0.34 (−2.13, 2.82)	0.79
	T1	106	13.1	(8.5, 18.7)	138	14.2	(9.5, 19.9)		
Minutes of LPA^c^	T0	171	74.0	18.7	180	73.4	20.7	*Descriptive analyses only*	
	T1	106	79.6	22.0	138	81.0	21.8		
Minutes of sedentary time^c^	T0	171	261.8	27.7	180	264.1	29.1	2.80 (−6.06, 11.66)	0.54
	T1	106	259.7	30.1	138	254.6	30.9		
Minutes of TPA on ECEC and non-ECEC days^c^	T0	88	74.0	28.6	122	72.6	27.9	Non-ECEC days: −2.14 (−13.81, 9.52)^f^	0.72
	T1	73	77.7	35.3	80	82.0	34.0	ECEC days: −3.86 (−12.90, 5.17)^f^	0.40
zBMI (UK90)	T0	240	0.47	0.94	266	0.50	0.95	0.015 (−0.207, 0.237)	0.89
	T1	159	0.57	1.06	204	0.47	1.00		
zBMI (WHO)^g^	T0	237	0.80	0.92	266	0.84	0.94	0.019 (−0.204, 0.242)	0.87
	T1	157	0.89	1.08	204	0.78	0.99		
% Overweight or obese^h^ (*n/N, %)*	T0	240	61/240	25.4%	266	69/266	25.9%	Adjusted OR 0.86 (0.52, 1.43)	0.56
	T1	159	42/159	26.4%	204	56/204	27.5%		

Abbreviations: ECEC, early childhood education and care; CI, confidence interval; SD, standard deviation; IQR, interquartile range; GMR, geometric mean ratio; MVPA, moderate/vigorous physical activity; LPA, light physical activity; zBMI, standardised body mass index.

a All models were adjusted for corresponding baseline outcome, child deprivation status and local authority area.

b Intraclass Correlation Coefficient (ICC) (95% CI) for ECEC: 0.130 (0.060, 0.260).

c Recorded during core ECEC hours between 9:00 and 15:00 h.

d ICC (95% CI) for ECEC: 0.072 (0.014, 0.303).

e Nutrition outcomes summarised as median (IQR).

f Interaction term: 1.72 (−9.18, 12.62), p = 0.76.

g Planned sensitivity analysis.

h Summarised as n/N (%).

Table 3 shows the results for all secondary outcomes. There were fewer kcals consumed and served at lunch in the intervention arm compared with the control arm at follow-up (aMD −67.7 (−118.6, −16.7), p = 0.009 for kcals consumed; aMD −69.1 (−116.0, −22.2), p = 0.004 for kcals served); median intakes consumed were within guidelines (359 kcal for lunch and 135 kcal for snacks^1^^,^^2^). The difference we saw in lunch serving and consumption (68 kcal) in the intervention arm represents a 19% reduction in portion size for lunch based upon an average of the recommended portion size (359 kcal) of children of this age group in England. The energy consumed from snacks was at the lower end of guidelines at baseline and there was no evidence the intervention changed kcal served or consumed for snacks. The intervention did not alter the nutritional quality of food (% kcal from non-core foods) in lunch or in snacks; or in minutes of MVPA, sedentary time, or TPA on ECEC and non-ECEC days. We did find at baseline that children were 20% more active on ECEC days compared with non-ECEC days (Appendix p8) and boys were 9.6% more active (TPA) than girls (Appendix p9). There was no evidence of a change in z-BMI or the proportion of children classified as overweight.

**Fig. 2:**
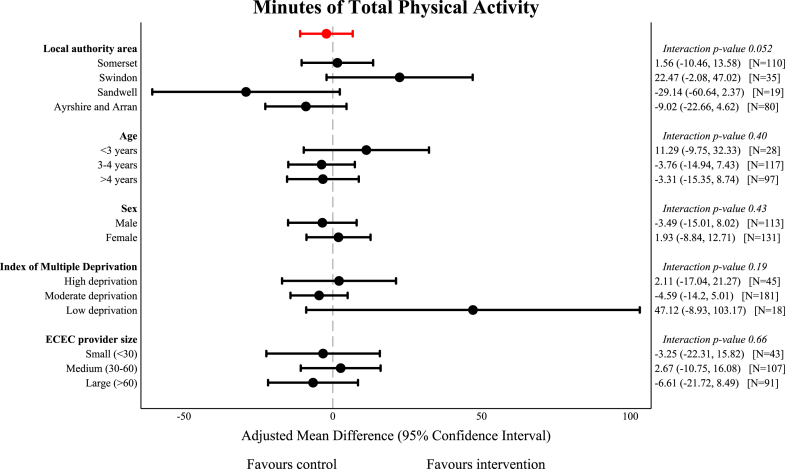
**Subgroup analysis for minutes of total physical activity**. Effect size from primary analysis shown in red. Abbreviations: ECEC, early childhood education and care.

**Fig. 3:**
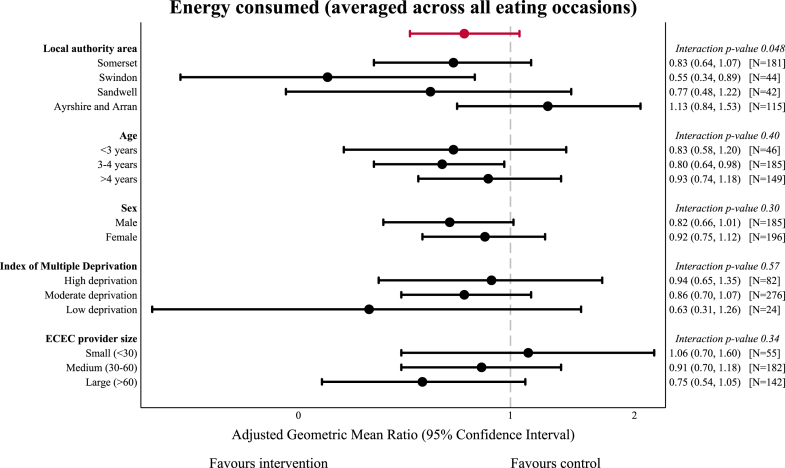
**Subgroup analysis for kcal energy consumed averaged across all eating occasions**. Effect size from primary analysis shown in red. Abbreviations: ECEC, early childhood education and care.

Figs. 2 and 3 show the subgroup analyses for the co-primary outcomes. There was no evidence of heterogeneity for the intervention effect for consumed kcal across any of the pre-specified subgroup analyses other than local authority area (interaction p-value 0.048). Consumed kcal averaged across all eating occasions were lower with the intervention compared with the control ECEC providers in Swindon, while there was no difference for ECEC providers in any other local authority (Appendix p10–14). There was no evidence of heterogeneity for the intervention effect for TPA across any of the subgroups except local authority area (interaction p-value 0.052), where there was no treatment effect for ECEC providers in Somerset and Ayrshire and Arran, weak evidence of increased minutes of TPA in Swindon (aMD 22.47 (−2.08, 47.02), p = 0.073), and weak evidence of lower TPA with the intervention in Sandwell (Appendix p15). These analyses were based on small numbers of children with considerable imbalance in the number of children within each treatment arm. Full summary tables are provided in Appendix p15.

Sensitivity analysis showed the effect estimates for the co-primary outcomes were robust to changes in model specification as per the pre-specified sensitivity analyses (Appendix p16); there was evidence of a reduction in kcal consumed across lunch and snacks after excluding outliers, and weak evidence of a reduction across all other sensitivity analyses. The results of additional post-hoc analyses are described in Appendix p17.

The process evaluation (reported in full separately^12^) assessed intervention implementation fidelity was generally high with 76% of intervention ECEC providers completing at least one cycle of NAPSACC UK (summary in Table 4). Learning about portion size was the most frequently reported learning by ECEC staff after the workshops (reported separately). Assessment of mediators found ECEC practitioners’ knowledge for child physical activity was higher in the intervention ECECs at follow-up with a median score of 81.2% (IQR 56.2%, 81.2%) in the intervention group; 65.6% (56.2%, 81.2%) in the control group) (Appendix p19). Motivation and self-efficacy to provide opportunity for physical activity were also slightly higher in the intervention ECEC providers. Both intervention and control providers had good knowledge of child nutrition at follow-up with a median score of 91.7% (84.4, 93.3) in the intervention group; 89.2% (77.5%, 94.4%) in the control group, and reported high levels of motivation and self-efficacy to provide nutritious food. Parental knowledge around physical activity, motivation, and self-efficacy to provide opportunity for physical activity were similar between the intervention and control groups at follow-up (see Appendix p20). Similarly, no differences in parental knowledge around nutrition, motivation, and self-efficacy to provide nutritious food were seen.

**Table 4:** Summary of NAPSACC UK process evaluation findings.^12^

Fidelity	• High fidelity for completing one cycle: 76% ECEC settings. • Moderate-to-low fidelity for completing two cycles: 40%
Partner training	• Training highly rated by Partners
Self-assessment	• Scores increased across all domains • Greater improvements for ECECs completing two cycles
Staff workshops	• Training highly rated by ECEC staff • In-person training preferred but pre-recorded training also highly acceptable
Goal setting	• 83% nutrition and 70% of physical activity goals were reported to have been fully or partially achieved • Policy changes hardest to implement
Tailored technical assistance	• Support highly valued by ECEC staff • Most assistance offered by email or in-person
Context	• Staffing shortages and time constraint delayed scheduling of staff workshops, preventing 10 ECECs from completing two full cycles • Most goals focused on increasing knowledge (staff, parent or child) which may not have translated into measurable impact on health outcomes • Both ECECs and Partners faced substantial sector-related pressures, limiting their capacity to fully engage with NAPSACC UK and implement more substantial changes

Abbreviation: ECEC, early childhood education and care.

## Discussion

Young children in ECEC settings exposed to the NAPSACC UK intervention did not have lower calorie intake averaged across eating occasion compared to children in those settings not exposed to the intervention, nor increased total physical activity during ECEC time. There was some evidence that the intervention led to improvements in the secondary outcomes of energy served and consumed at lunch. We saw no evidence of change in any of the other measures of physical activity, nutrition or change in measures of adiposity. NAPSACC has been widely adopted in the US across 22 states with over 7000 ECEC reaching over 344,000 children using online delivery called ‘Go NAPSACC’.^25^ It has been assessed to have the strongest evidence to reduce child obesity risk in the US.^26^ However, our trial findings do not support the roll-out of the adapted NAPSACC in the UK, where there are contextual differences compared to the US. The heterogeneity of the context of ECECs settings within countries and between countries, such as funding, regulations, standards, policies, practices, training, indoor and outdoor environments, weather, food provision and child attendance, make international intervention comparisons challenging. The lower fidelity to the intervention than intended and context of staffing pressures give insight into interpretation of our study's null result. This is explored in more detail in the separately reported process evaluation.^12^

Reviews of other trials which aim to improve diet and in ECECs have found uncertain evidence of possible improvement in child diet quality.^5^ Meal size is potentially critical for maintaining a healthy weight, with each additional 10 kcal consumed per meal associated with a 7% faster rate of weight gain in 2–5 year-old children.^27^ A systematic review of experimental studies which increased child portion size found increased consumption.^28^ Whilst reducing energy consumed may not always be the most appropriate objective for children, the aim of NAPSACC UK was to do this within nationally recommended levels. In our study, the median serving size of lunches exceeded portion size guidelines^29^^,^^30^ for this age group and was higher in lunchboxes than provided by ECEC settings. In contrast, the portion sizes served and consumed for snacks were lower than national guidelines at baseline, suggesting it was appropriate that energy served did not change in response to the NAPSACC UK intervention. There was variability in UK ECEC food provision as Scotland had national ECEC food standards, free ECEC lunch provision and correspondingly almost no lunch boxes.^31^ In contrast, in English ECECs there were no national mandated food standards at the time of the study, limited free lunch provision, and half the children in England in our study had parent-provided lunchboxes. This intervention did not change parental nutritional mediators of knowledge, motivation or self-efficacy which highlights the limitations of ECEC focused interventions to change lunchbox food quality and content. Systematic reviews of the small number of ECEC lunchbox specific interventions have found limitations with these approaches including low parent engagement, difficulties with sustainability, and resource-intensive strategies making scale-up challenging.^32^

The null results in this trial for all measures of physical activity are explored in the process evaluation (separately reported).^12^ Our findings are consistent with the findings of a systematic review of strategies, policies, practices, or programmes to improve physical activity within ECEC services.^8^ A meta-analysis^33^ of ECEC physical activity interventions found small increases in physical activity, however the two UK full trials in this review did not find evidence for increases in physical activity and the third was a feasibility study. The null results for physical activity found in our trial compared with the findings of potential for promise seen in our feasibility study are likely to be in part due to the commonly experienced generalisability biases that are evident when scaling up from a pilot study to a definitive trial, with delivery agent bias being identified as often a key difference between pilot and definitive trials.^34^ The low levels of activity we observed are consistent with other studies.^3^ While the thresholds we used for determining total physical activity and MVPA were on the higher side, our findings show high levels of sedentary behaviour and are consistent with findings in other studies that children are a long way off reaching recommended levels.^3^ The higher PA levels we observed for children on ECEC days compared to non-ECEC days highlights the importance of these settings for physical activity. Our findings are consistent with the structured days hypothesis, that pre-planned, segmented activities with adult supervision can increase physical activity.^35^ The differences observed between boys and girls activity on ECEC days is consistent with the well documented higher levels in boys than girls at young ages,^3^ however the reasons for this at an ECEC level need further exploration.

In terms of strengths, our research team was independent from the intervention delivery team, random allocations were only revealed to ECEC after baseline data had been collected and our measurements were objective. All outcomes were assessed using age-appropriate, outcome measures including the novel use of food photography to estimate portion size served and consumed in ECEC providers. We achieved recruitment of a diverse group of ECEC providers, across four areas of the UK and recruitment of a group of children with varied deprivation and ethnicity (14% non-white). Despite numerous contextual challenges at a societal level in the UK and the ECEC sector during the period of the study (Appendix p6) the intervention was delivered with fidelity, albeit at a lower dose than intended.

In terms of limitations, although ECEC dropout could have introduced bias, attrition rates were similar between groups, resulting in an unclear influence on effectiveness but a probable loss of precision in the effect estimates. The main limitation of the study was with the well documented national ECEC sector cost and staffing pressures (Appendix p6). These challenges led to delays in scheduling staff workshops with only 40% ECEC able to complete two intervention cycles. Related to this, to minimise participant burden we did not collect data on the stability of the ECEC workforce for the duration of the intervention or the proportion of the workforce who engaged with the training. The study had greater heterogeneity in food provision, with different ECEC nutrition guidelines, standards and provision by country, and with more lunchboxes than anticipated from the feasibility trial. However, this heterogeneity has provided insight into opportunities for policy changes. In keeping with other public health community-based studies, we were not able to blind ECEC providers to study arm. The repeated cross-sectional design and lack of allocation concealment also has limitations due to the potential for selection bias with enrolment at T1, however we did not see large differences in the numbers of children either leaving the study between T0 and T1, or joining the study before data collection at T1, between the two arms; demography data were compared between groups at both T0 and T1 and analyses were adjusted to account for any imbalances observed. A further limitation of our study with respect to the analysis of PA data is the high proportion of children for whom valid activity data were not available. A high level of missingness was anticipated due to the nature of the outcome assessment, and our level of missing PA outcome data at both baseline and follow-up are in line with expectations (∼35%) and balanced across arms, therefore no imputation of missing data was carried out. Reasons for missing outcome data from accelerometers include children not attending the ECEC setting for a minimum of two days in the week of data collection (despite their usual pattern of attendance deeming them eligible) and children not wearing accelerometers for a sufficient period of time throughout the day. We would recommend strategies to maximise the level of valid data in future studies of PA, particularly studies in this young age group.

Further research is needed to understand the barriers and opportunities to improve nutrition and PA in ECEC settings in the UK, including the role of policy, regulation and statutory changes.^1^ ECEC setting-based interventions require high agency (conscious individual action),^36^ local coordination and investment. The UK context of restricted public health funding and constrained ECEC sector capacity suggests that policy-level and statutory changes, which require low agency by individual ECEC settings may be more fruitful endeavours. This approach is further supported by the lack of evidence from interventions to improve lunchboxes in ECECs beyond improvements in vegetable servings.^37^ Mandated nutritional values and portion sizes, with free provision of lunches, as provided in ECEC in Scotland and in infant schools in England,^38^ may provide the greatest potential to have comprehensive reach and reduction in health inequalities. It is a priority to evaluate the acceptability and impact of new 2025 Government nutrition guidance for ECEC settings in England,^39^ particularly given the focus of the guidance on ECEC provided food rather than lunchbox provision. The recent review^33^ providing evidence of effective ECEC interventions to increase physical activity in countries other than the UK, should inform the design and delivery of PA policy or interventions in ECECs in the UK and other countries. In conclusion, our recommendation is that the priority for research and policy is to consider the provision, acceptability, and value of policy and statutory changes for nutrition and PA in ECEC.

## Contributors

The study was conceived by RK, MP, CM, RJ, LJ, RL, CM, WH, JW, LM and SS. KH and KW were the Trial Managers, MC was the health economics analyst, RM was the trial statistician. JT was the trials centre lead and PB was the senior trial statistician, ZT and LJ were the nutrition co-leads. The first draft of this manuscript was written by RK, KH, and RM with input from all other authors. RM, PB, CM, RK, KH had access to the raw and verified data. RM and KH take responsibility for the integrity of the data. RM and PB take responsibility for the accuracy of the data analysis. All authors have edited and critically reviewed the paper and approved the final version of the paper. All authors take responsibility for the decision to submit the paper for publication.

## Data sharing statement

Data for the NAPSACC UK study is available upon request after approval with a signed data access agreement (https://data.bris.ac.uk/data/). The study protocol was published in the BMC public health and is available at: https://bmcpublichealth.biomedcentral.com/articles/10.1186/s12889-023-16229-y. The statistical analysis plan is available at: (Nutrition and Physical Activity Self-Assessment for Child Care (NAP SACC UK Trial): Statistical Analysis Plan–University of Bristol) and the health economics analysis plan is available at: (HEALTH ECONOMICS ANALYSIS PLAN (HEAP) NAP SACC UK–University of Bristol).

## Declaration of interests

R.J. has received Institutional grant payments from National Institute of Health and Care Research (NIHR) UK. These were the payments that funded this specific project as well as support via NIHR Applied Research Collaboration (ARC) West and Bristol Biomedical Research Centre (Bristol BRC). J.W. has received grant payments from NIHR and Health and Care Research Wales. L.J. has received grant payments from NIHR and Wellcome Trust and has received consulting fees from Nesta for participating in an Expert Advisory Group (https://blueprint.nesta.org.uk/). M.M has received grant payments from NIHR. SAS has had unpaid leadership roles within UK Society of Behavioral Medicine, HTA Clinical Evaluation and Trials Committee, Commissioning panel for NIHR program and Chief Scientific Office HIPS Committee. W.H. and M.C. have received grant payments from NIHR. M.P. has received grant payments from NIHR and has participated as a member of the data monitoring and ethics committee for the NIHR funded HENRY III Trial. S.C. has received Institutional funding from Scottish Government Health and Social Care Directorates and the Medical Research Council. C.K.M has received grant payments from NIHR. L.M. has received Institutional grant payments from NIHR. C.M. has received grant funding from NIHR for this work.

## Appendix A Supplementary data

Supplementary Figures and Tables

Protocol
