# Supplementary material for: Effectiveness of an environmental nutrition and physical activity intervention in early childhood education and care settings (NAPSACC UK): a multicentre cluster randomised controlled trial
Source: Lancet Reg Health Eur. 2025 Dec 18;61:101550. doi: 10.1016/j.lanepe.2025.101550 (PMC12882655; doi:10.1016/j.lanepe.2025.101550)
Supplement: Supplementary Figures and Tables [file mmc1.docx]

**Effectiveness of an environmental nutrition and physical activity intervention in early childhood education and care settings: a multicentre cluster randomised controlled trial (NAPSACC UK)**

**Supplementary material**

Contents

[Figure S1: NAPSACC UK Intervention Cycle 2](#_Toc214975039)

[Figure S2 Logic Model: NAPSACC UK 3](#_Toc214975040)

[Figure S3: Statistical methods - additional information 4](#_Toc214975041)

[Table S1: Sensitivity analyses 5](#_Toc214975042)

[Figure S4: Major contextual changes occurring during trial 6](#_Toc214975043)

[Table S2: Demography table (ECEC provider characteristics) 7](#_Toc214975044)

[Table S3: Physical activity data summarised across all children with activity data at T0, summarised separately by day type 8](#_Toc214975045)

[Table S4: Total Physical Activity (TPA) on ECEC days by sex 9](#_Toc214975046)

[Table S5: Energy (kcal) consumed subgroup analyses 10](#_Toc214975047)

[Table S6: Total Physical Activity subgroup analyses 15](#_Toc214975048)

[Table S7: Sensitivity analyses 16](#_Toc214975049)

[Figure S5: Results from post-hoc nutrition analyses 17](#_Toc214975050)

[Table S8: kcal consumed, kcal served and % kcal from non-core food summarised by type of food provision for lunches (including England and Scotland) 18](#_Toc214975051)

[Table S9: ECEC provider mediator questionnaires[9] 19](#_Toc214975052)

[Table S10: Parent Mediator questionnaires[9] 20](#_Toc214975053)

[Table S11: Review and Reflect Tool 21](#_Toc214975054)

[**References** 62](#_Toc214975055)

## Figure S1: NAPSACC UK Intervention Cycle


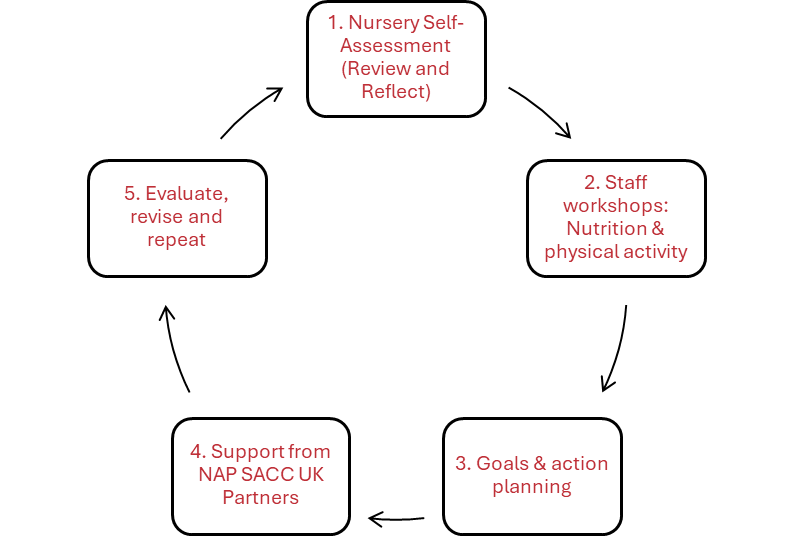


## Figure S2 Logic Model: NAPSACC UK


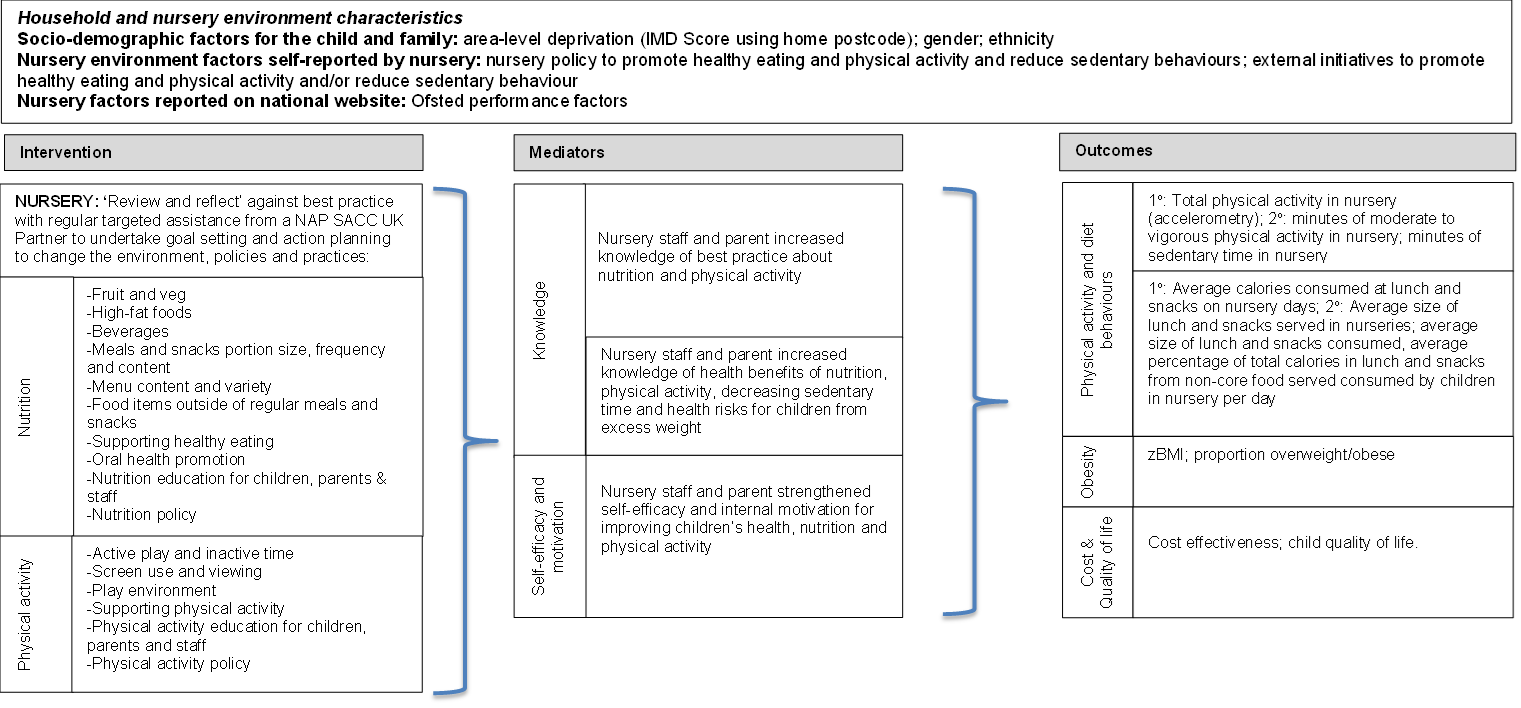


## Figure S3: Statistical methods - additional information

*Processing of activity data*

Prior to analyses, activity data were cleaned and periods of non-wear time removed (identified as period of 60 minutes or more with zero values). For each 10-second interval, activity levels were classified using the cut-points proposed by Puyau et al[3].: sedentary time (ST) 0-200 counts/15 second epoch; light physical activity (LPA) 200-799 counts/15 second epoch; and moderate/vigorous physical activity (MVPA) ≥800 counts/15 second epoch. For each day, the total time (in minutes) spent in each type of activity (ST, LPA, MVPA, TPA; TPA=LPA+MVPA) and total wear time wear time were calculated. Each weekday with physical activity was classified as an ECEC weekday (attendance at ECEC setting for a minimum of 3 hours) or non-ECEC weekday (no attendance at an ECEC setting); any days with ECEC attendance <3 hours were excluded. The mean number of minutes spent in each of activity were calculated across ECEC and non-ECEC weekdays; a minimum of 2 days were required for calculation of summary statistics over ECEC days. Activity data were summarised during core ECEC provider hours (09:00-15:00)[4], with a minimum of four hours wear time for the day to be included in summary measures. Analyses were repeated summarising data across the whole recording period (06:00-00:00), with a requirement of at least six hours wear time for inclusion in summary measures.

*Derivation of other outcome measures*

z-BMI was calculated according to the UK 1990 growth reference chart and the WHO reference chart. Z-BMI (1990) was categorised into healthy weight (<85^th^ centile, zBMI<1.04), overweight (≥85th centile, zBMI≥1.04) and obese (≥95^th^ centile, zBMI≥1.64). Deprivation level was categorised into high (deciles 1-3 using IMD or SIMD), medium (deciles 4-7) and low (deciles 8-10).

## Table S1: Sensitivity analyses

| Sensitivity analysis | Outcome | Details |
| --- | --- | --- |
| Compliance | Co-primary activity and nutrition outcomes | A pre-specified sensitivity analysis to investigate the effect of the NAPSACC UK intervention excluding those intervention nurseries that did not engage with the NAPSACC UK intervention.  Primary outcome models were refitted excluding children at ECEC providers who did not comply with the intervention. Compliance with the intervention was assessed at two levels.  Level 1:   1. - One cycle of self-assessment (called Review & Reflect) completed 2. - Engagement from at least one member of ECEC staff in one of the workshops either in-person or online 3. - One cycle of goal setting completed   Level 2:   1. - Two cycles of self-assessment (called Review & Reflect) completed 2. - Engagement from at least one member of ECEC staff in one of the workshops either in-person or online   - Two cycles of goal setting completed |
| Additional covariates | Co-primary activity and nutrition outcomes | A pre-specified sensitivity analysis to adjust for additional covariates that were not balanced between groups at baseline. |
| Outliers | Co-primary activity and nutrition outcomes | A pre-specified sensitivity analysis excluding data points identified as outliers on graphical checks of model fit. |
| Growth reference charts | zBMI | As specified, the secondary outcome zBMI was performed for zBMI calculated using UK90 age and sex reference charts. A pre-specified sensitivity analysis on the outcome zBMI calculated using the WHO growth reference charts was also performed. |
| Seasonality | Co-primary activity and nutrition outcomes | Data collection T0 and T1 spanned 10 and 7 months, respectively. Due to the anticipated difference in opportunities for physical activity and variability in foods typically eaten across seasons, post-hoc sensitivity analyses adjusting for the hours of daylight on the day of data collection were performed (day of RFPM for the nutrition outcome, day of accelerometer issue for the physical activity outcome. |

Abbreviations: ECEC – early childhood education and care; zBMI – standardised body mass index; RFPM – remote food photography method.

## Figure S4: Major contextual changes occurring during trial

- COVID-19 global pandemic: The coronavirus global pandemic started in 2020 and increased financial pressures on providers and instability in the childcare provider sector. The pandemic also exacerbated existing staffing issues with recruitment and retainment retention of staff, particularly highly-qualified staff.[1]

- ‘Brexit’: The UK’s decision to leave the European Union came into effect in 2020 and led to increased difficulty in recruiting new staff across the social care sector.[2]

- Cost of living: The UK ‘cost of living crisis’ started in 2021 and refers to a fall in average household disposable incomes, meaning that the cost of everyday household essentials such as energy and grocery bills are increasing at a faster rate than income.[5] Since 2021 there has been an increase in the percentage of families experiencing difficulties meeting childcare costs.[6]
- Increase in Government-funded free childcare hours in England and Scotland: In England, The entitlement of 30 free hours childcare for three-to-four year olds for eligible parents (those that earn less than £100,000 a year each) is being expanded to include children from nine months old in phases from April 2024 onwards with full entitlement in place from September 2025[1]. In Scotland, the amount of funded childcare for three-to-four year olds and eligible two-year olds was expanded from 600 hours to 1,140 hours in August 2021 (delayed from August 2020 due to the pandemic)[7].
- Increased closure of childcare providers: Since 2015, there has been a steady decrease in the number of childcare providers in England[1]. From 2022 to 2023 there was an estimated 5% decrease in the total number of providers, and a 10% decline in the number of childminders.[8]

## Table S2: Demography table (ECEC provider characteristics)

|  | | Intervention ECEC providers  N=25 | | Control ECEC providers  N=27 | |
| --- | --- | --- | --- | --- | --- |
| ECEC deprivation status (a) | |  |  |  |  |
|  | High deprivation | 7/25 | 28.0% | 6/27 | 22.2% |
|  | Moderate deprivation | 15/25 | 60.0% | 19/27 | 70.4% |
|  | Low deprivation | 3/25 | 12.0% | 2/27 | 7.4% |
| Local authority area | |  |  |  |  |
|  | Somerset | 9/25 | 36.0% | 10/27 | 37.0% |
|  | Swindon | 4/25 | 16.0% | 5/27 | 18.5% |
|  | Sandwell | 5/25 | 20.0% | 4/27 | 14.8% |
|  | Ayrshire and Arran | 7/25 | 28.0% | 8/27 | 29.6% |
| ECEC provider size | |  |  |  |  |
|  | Small (<30 children) | 6/23 | 26.1% | 5/27 | 18.5% |
|  | Medium (30-60 children) | 11/23 | 47.8% | 14/27 | 51.9% |
|  | Large (>60 children) | 6/23 | 26.1% | 8/27 | 29.6% |
| ECEC provider type | |  |  |  |  |
|  | Nursery attached to school | 4/25 | 16.0% | 6/27 | 22.2% |
|  | Nursery not attached to school | 9/25 | 36.0% | 10/27 | 37.0% |
|  | Pre-school attached to school | 7/25 | 28.0% | 4/27 | 14.8% |
|  | Pre-school not attached to school | 5/25 | 20.0% | 7/27 | 25.9% |

Abbreviations: ECEC – early childhood education and care. Note: Deprivation status calculated using IMD (2019) or SIMD (2020), categorised into high deprivation (decile 1-3), moderate deprivation (decile 4-7) or low deprivation (decile 8-10).

a Based on children with data collection at either T0 or T1

## Table S3: Physical activity data summarised across all children with activity data at T0, summarised separately by day type

|  | | T0 | | |
| --- | --- | --- | --- | --- |
|  | | N | Mean | SD |
| Mean wear time (minutes) | |  |  |  |
|  | Nursery day | 351 | 350.6 | 19.2 |
|  | Non-nursery weekday | 210 | 343.9 | 29.1 |
|  | Weekend day | 100 | 332.0 | 35.4 |
| Total Physical Activity (minutes) | |  |  |  |
|  | Nursery day | 351 | 87.6 | 25.9 |
|  | Non-nursery weekday | 210 | 73.2 | 28.1 |
|  | Weekend day | 100 | 80.4 | 30.9 |
| Moderate/Vigorous Physical Activity (minutes) (median, IQR) | |  |  |  |
|  | Nursery day | 351 | 12.9 | (7.9, 17.6) |
|  | Non-nursery weekday | 210 | 9.4 | (5.8, 14.8) |
|  | Weekend day | 100 | 11.6 | (7.0, 16.8) |
| Light Physical Activity (minutes) | |  |  |  |
|  | Nursery day | 351 | 73.7 | 19.7 |
|  | Non-nursery weekday | 210 | 61.8 | 22.2 |
|  | Weekend day | 100 | 66.5 | 23.7 |
| Sedentary time (minutes) | |  |  |  |
|  | Nursery day | 351 | 263.0 | 28.4 |
|  | Non-nursery weekday | 210 | 270.7 | 34.2 |
|  | Weekend day | 100 | 251.6 | 37.7 |

Abbreviations: SD – standard deviation; IQR – interquartile range.

Data are summarised between 9am and 3pm. Lower numbers of children provided physical activity data at weekends; activity belts were programmed to record for a period of 7 consecutive days, however parents were asked to put activity belts on children for weekdays with no guidance over weekend wear. Consequently, only a small proportion of children wore activity belts over the weekend, hence the lower number of children included in the analyses of weekend day data.

## Table S4: Total Physical Activity (TPA) on ECEC days by sex

|  | Male | | | Female | | | Overall | | |
| --- | --- | --- | --- | --- | --- | --- | --- | --- | --- |
|  | N | Mean | SD | N | Mean | SD | N | Mean | SD |
| Minutes of TPA at T0 | 175 | 154.7 | 43.4 | 175 | 141.2 | 40.9 | 350 | 147.9 | 42.6 |
| Minutes of TPA at T1 | 113 | 165.9 | 47.1 | 135 | 146.4 | 38.0 | 248 | 155.3 | 43.4 |
|  | N | n | % | N | n | % | N | n | % |
| Meets CMO 180 min TPA guidelines (T0) | 175 | 47 | 26.9% | 175 | 34 | 19.4% | 350 | 81 | 23.1% |
| Meets CMO 180 min TPA guidelines (T1) | 113 | 44 | 38.9% | 135 | 26 | 19.3% | 248 | 70 | 28.2% |
| Meets CMO 60 min MVPA guidelines (T0) | 143 | 5 | 3.5% | 151 | 2 | 1.3% | 294 | 7 | 2.4% |
| Meets CMO 60 min MVPA guidelines (T1) | 99 | 2 | 2.0% | 118 | 1 | 0.9% | 217 | 3 | 1.4% |

Abbreviations: SD – standard deviation; TPA – Total Physical Activity; CMO – Chief Medical Officer; MVPA – moderate/vigorous physical activity.

Data are summarised over the full day, not restricted to 9am to 3pm as per the primary analyses.

## Table S5: Energy (kcal) consumed subgroup analyses

|  | Eating occasion | Data period |  | Intervention ECEC providers | | | Control ECEC providers | | | Adjusted Geometric Mean Ratio  (95% CI), p-value |
| --- | --- | --- | --- | --- | --- | --- | --- | --- | --- | --- |
|  |  |  |  | N | Median | IQR | N | Median | IQR |  |
| Local authority area | |  |  |  |  |  |  |  |  | *Interaction p-value 0.048* |
| Somerset (n=181) | Lunch | T0 |  | 107 | 370.2 | (229.2, 489.4) | 110 | 344.1 | (228.3, 455.5) | 0.83 (0.64, 1.07) |
|  |  | T1 |  | 87 | 404.0 | (259.0, 493.0) | 94 | 443.4 | (312.3, 586.6) |  |
|  | Morning snack | T0 |  | 101 | 86.0 | (51.0, 129.6) | 97 | 95.0 | (36.5, 181.8) |  |
|  |  | T1 |  | 83 | 56.5 | (37.0, 103.6) | 83 | 80.2 | (37.0, 169.3) |  |
|  | Afternoon snack | T0 |  | 80 | 87.0 | (46.1, 218.7) | 55 | 137.8 | (84.5, 170.3) |  |
|  |  | T1 |  | 45 | 62.5 | (27.0, 93.7) | 53 | 89.7 | (45.2, 130.5) |  |
| Swindon (n=44) | Lunch | T0 |  | 20 | 425.8 | (260.1, 484.5) | 43 | 385.4 | (257.0, 462.6) | 0.55 (0.34, 0.89) |
|  |  | T1 |  | 10 | 235.0 | (95.0, 281.6) | 34 | 331.6 | (224.8, 507.3) |  |
|  | Morning snack | T0 |  | 19 | 106.3 | (52.6, 183.8) | 39 | 70.7 | (29.5, 95.0) |  |
|  |  | T1 |  | 10 | 77.6 | (12.4, 129.3) | 26 | 75.9 | (31.1, 114.0) |  |
|  | Afternoon snack | T0 |  | 6 | 156.3 | (22.8, 183.5) | 9 | 93.8 | (80.1, 116.0) |  |
|  |  | T1 |  | 7 | 115.4 | (81.4, 115.4) | 5 | 95.2 | (87.2, 98.5) |  |
| Sandwell (n=42) | Lunch | T0 |  | 42 | 225.8 | (144.8, 374.7) | 37 | 331.2 | (176.0, 449.6) | 0.77 (0.48, 1.22) |
|  |  | T1 |  | 28 | 238.8 | (148.4, 317.4) | 14 | 399.3 | (269.2, 513.8) |  |
|  | Morning snack | T0 |  | 40 | 45.4 | (20.7, 103.2) | 37 | 56.3 | (28.1, 93.0) |  |
|  |  | T1 |  | 28 | 107.7 | (47.6, 182.8) | 13 | 105.3 | (72.9, 129.0) |  |
|  | Afternoon snack | T0 |  | 30 | 67.6 | (51.0, 138.7) | 36 | 26.1 | (14.7, 48.7) |  |
|  |  | T1 |  | 26 | 109.4 | (56.4, 155.6) | 12 | 45.2 | (24.2, 101.7) |  |
| Ayrshire and Arran (n=115) | Lunch | T0 |  | 92 | 287.2 | (208.4, 386.6) | 81 | 292.9 | (199.5, 365.4) | 1.13 (0.84, 1.53) |
|  |  | T1 |  | 47 | 323.3 | (210.4, 403.3) | 68 | 327.2 | (197.0, 436.6) |  |
|  | Morning snack | T0 |  | 92 | 64.4 | (32.2, 123.4) | 78 | 55.5 | (22.0, 122.3) |  |
|  |  | T1 |  | 45 | 58.1 | (28.2, 126.9) | 52 | 59.7 | (26.6, 117.8) |  |
|  | Afternoon snack | T0 |  | 10 | 44.3 | (33.7, 76.2) | 19 | 202.2 | (73.1, 320.4) |  |
|  |  | T1 |  | 2 | 67.3 | (60.1, 74.5) | 8 | 95.7 | (83.8, 254.2) |  |
| Country | |  |  |  |  |  |  |  |  | *Interaction p-value 0.042* |
| England (n=267) | Lunch | T0 |  | 169 | 333.6 | (212.1, 450.8) | 190 | 344.1 | (222.8, 454.9) | 0.79 (0.64, 0.97) |
|  |  | T1 |  | 125 | 350.6 | (202.4, 448.5) | 142 | 418.7 | (269.2, 577.0) |  |
|  | Morning snack | T0 |  | 160 | 78.1 | (35.5, 129.6) | 173 | 70.7 | (33.9, 137.3) |  |
|  |  | T1 |  | 121 | 61.4 | (37.0, 114.7) | 122 | 80.2 | (37.5, 132.0) |  |
|  | Afternoon snack | T0 |  | 116 | 87.0 | (46.1, 192.2) | 100 | 85.5 | (23.7, 147.2) |  |
|  |  | T1 |  | 78 | 76.2 | (49.6, 132.5) | 70 | 83.3 | (36.9, 123.1) |  |
| Scotland (n=115) | Lunch | T0 |  | 92 | 287.2 | (208.4, 386.6) | 81 | 292.9 | (199.5, 365.4) | 1.14 (0.83, 1.55) |
|  |  | T1 |  | 47 | 323.3 | (210.4, 403.3) | 68 | 327.2 | (197.0, 436.6) |  |
|  | Morning snack | T0 |  | 92 | 64.4 | (32.2, 123.4) | 78 | 55.5 | (22.0, 122.3) |  |
|  |  | T1 |  | 45 | 58.1 | (28.2, 126.9) | 52 | 59.7 | (26.6, 117.8) |  |
|  | Afternoon snack | T0 |  | 10 | 44.3 | (33.7, 76.2) | 19 | 202.2 | (73.1, 320.4) |  |
|  |  | T1 |  | 2 | 67.3 | (60.1, 74.5) | 8 | 95.7 | (83.8, 254.2) |  |
| Age | |  |  |  |  |  |  |  |  | *Interaction p-value 0.40* |
| <3 years (n=46) | Lunch | T0 |  | 42 | 320.8 | (196.8, 446.1) | 58 | 299.4 | (231.1, 415.5) | 0.83 (0.58, 1.20) |
|  |  | T1 |  | 28 | 393.8 | (206.2, 494.9) | 18 | 309.8 | (229.9, 529.1) |  |
|  | Morning snack | T0 |  | 41 | 69.6 | (46.3, 117.8) | 54 | 68.0 | (32.3, 162.5) |  |
|  |  | T1 |  | 27 | 47.5 | (24.5, 88.0) | 16 | 86.6 | (61.7, 228.7) |  |
|  | Afternoon snack | T0 |  | 30 | 75.5 | (23.0, 252.8) | 32 | 146.7 | (34.3, 182.0) |  |
|  |  | T1 |  | 12 | 71.0 | (59.4, 91.2) | 11 | 87.2 | (45.2, 131.8) |  |
| 3-4 years (n=185) | Lunch | T0 |  | 141 | 316.6 | (217.4, 439.4) | 123 | 305.3 | (201.6, 445.9) | 0.80 (0.64, 0.98) |
|  |  | T1 |  | 82 | 298.1 | (176.3, 435.6) | 103 | 395.5 | (263.8, 535.4) |  |
|  | Morning snack | T0 |  | 136 | 71.9 | (33.1, 128.2) | 117 | 62.0 | (30.9, 112.0) |  |
|  |  | T1 |  | 79 | 76.5 | (46.4, 119.3) | 88 | 80.5 | (37.0, 140.7) |  |
|  | Afternoon snack | T0 |  | 59 | 70.6 | (43.2, 130.0) | 44 | 85.0 | (16.0, 145.3) |  |
|  |  | T1 |  | 47 | 85.6 | (50.9, 147.3) | 43 | 89.7 | (45.8, 119.3) |  |
| >4 years (n=149) | Lunch | T0 |  | 76 | 325.1 | (229.4, 420.6) | 89 | 348.1 | (238.6, 439.0) | 0.93 (0.74, 1.18) |
|  |  | T1 |  | 60 | 344.5 | (225.8, 415.1) | 89 | 361.7 | (234.8, 503.3) |  |
|  | Morning snack | T0 |  | 73 | 85.1 | (32.9, 131.4) | 80 | 70.0 | (28.5, 129.5) |  |
|  |  | T1 |  | 58 | 52.4 | (28.2, 118.7) | 70 | 66.2 | (28.1, 117.4) |  |
|  | Afternoon snack | T0 |  | 37 | 127.5 | (64.8, 220.4) | 42 | 96.2 | (21.7, 141.1) |  |
|  |  | T1 |  | 19 | 60.1 | (16.5, 108.9) | 24 | 88.3 | (35.3, 153.7) |  |
| Sex | |  |  |  |  |  |  |  |  | *Interaction p-value 0.30* |
| Male (n=185) | Lunch | T0 |  | 119 | 298.4 | (217.4, 460.0) | 145 | 301.1 | (215.5, 437.8) | 0.82 (0.66, 1.01) |
|  |  | T1 |  | 78 | 328.6 | (217.0, 480.0) | 107 | 418.2 | (255.6, 529.1) |  |
|  | Morning snack | T0 |  | 115 | 78.0 | (35.7, 139.2) | 133 | 62.0 | (30.4, 136.1) |  |
|  |  | T1 |  | 74 | 60.6 | (29.5, 116.0) | 92 | 76.0 | (39.1, 128.7) |  |
|  | Afternoon snack | T0 |  | 58 | 80.7 | (35.6, 202.4) | 59 | 73.6 | (10.2, 148.1) |  |
|  |  | T1 |  | 35 | 60.5 | (37.0, 132.5) | 44 | 94.4 | (48.7, 141.5) |  |
| Female (n=196) | Lunch | T0 |  | 140 | 333.6 | (209.5, 431.5) | 125 | 336.6 | (219.8, 437.8) | 0.92 (0.75, 1.12) |
|  |  | T1 |  | 93 | 344.1 | (210.4, 413.5) | 103 | 357.1 | (231.9, 503.3) |  |
|  | Morning snack | T0 |  | 135 | 68.2 | (33.3, 123.7) | 117 | 67.7 | (30.5, 122.2) |  |
|  |  | T1 |  | 91 | 68.2 | (30.7, 118.7) | 82 | 77.0 | (32.0, 134.0) |  |
|  | Afternoon snack | T0 |  | 68 | 82.7 | (46.2, 158.9) | 60 | 107.8 | (26.9, 156.9) |  |
|  |  | T1 |  | 45 | 88.6 | (60.5, 131.0) | 34 | 79.1 | (29.9, 119.3) |  |
| Child deprivation status | |  |  |  |  |  |  |  |  | *Interaction p-value 0.57* |
| High deprivation (n=82) | Lunch | T0 |  | 70 | 314.0 | (180.3, 400.0) | 56 | 260.4 | (182.1, 422.3) | 0.94 (0.65, 1.35) |
|  |  | T1 |  | 53 | 293.9 | (182.1, 390.9) | 29 | 311.0 | (209.3, 478.4) |  |
|  | Morning snack | T0 |  | 67 | 35.7 | (18.7, 68.2) | 47 | 69.2 | (38.2, 112.6) |  |
|  |  | T1 |  | 51 | 50.2 | (28.2, 93.9) | 20 | 90.9 | (44.8, 130.0) |  |
|  | Afternoon snack | T0 |  | 34 | 49.4 | (31.1, 82.1) | 42 | 25.9 | (10.2, 55.4) |  |
|  |  | T1 |  | 34 | 71.1 | (44.0, 109.8) | 13 | 53.4 | (18.5, 97.8) |  |
| Moderate deprivation (n=276) | Lunch | T0 |  | 165 | 317.3 | (215.0, 443.7) | 192 | 317.5 | (219.9, 427.1) | 0.86 (0.70, 1.07) |
|  |  | T1 |  | 115 | 362.4 | (230.3, 462.7) | 161 | 366.9 | (245.9, 514.7) |  |
|  | Morning snack | T0 |  | 160 | 88.2 | (47.5, 146.9) | 182 | 67.2 | (28.1, 134.9) |  |
|  |  | T1 |  | 111 | 68.2 | (44.2, 121.9) | 134 | 78.1 | (37.5, 159.5) |  |
|  | Afternoon snack | T0 |  | 77 | 83.3 | (47.5, 180.7) | 59 | 147.1 | (82.2, 219.6) |  |
|  |  | T1 |  | 42 | 84.9 | (56.4, 155.6) | 51 | 95.7 | (45.8, 151.3) |  |
| Low deprivation (n=24) | Lunch | T0 |  | 26 | 382.0 | (234.2, 590.6) | 23 | 452.1 | (348.1, 506.8) | 0.63 (0.31, 1.26) |
|  |  | T1 |  | 4 | 217.1 | (162.0, 266.7) | 20 | 418.6 | (314.7, 577.3) |  |
|  | Morning snack | T0 |  | 25 | 117.8 | (70.0, 180.7) | 22 | 51.2 | (43.5, 119.5) |  |
|  |  | T1 |  | 4 | 64.8 | (13.0, 144.4) | 20 | 58.4 | (31.1, 89.1) |  |
|  | Afternoon snack | T0 |  | 15 | 202.4 | (156.3, 259.8) | 18 | 139.4 | (43.8, 152.0) |  |
|  |  | T1 |  | 4 | 66.1 | (50.9, 83.5) | 14 | 72.1 | (45.2, 122.9) |  |
| ECEC provider size | |  |  |  |  |  |  |  |  | *Interaction p-value 0.34* |
| Small (n=55) | Lunch | T0 |  | 45 | 316.6 | (231.5, 443.7) | 38 | 309.9 | (223.1, 419.9) | 1.06 (0.70, 1.60) |
|  |  | T1 |  | 25 | 288.4 | (199.8, 396.7) | 30 | 361.3 | (290.2, 491.2) |  |
|  | Morning snack | T0 |  | 45 | 114.7 | (51.3, 183.8) | 35 | 46.1 | (8.9, 95.0) |  |
|  |  | T1 |  | 25 | 130.3 | (107.3, 156.0) | 29 | 73.3 | (28.1, 121.5) |  |
|  | Afternoon snack | T0 |  | 16 | 58.0 | (36.6, 75.0) | 2 | 36.6 | (0.0, 73.1) |  |
|  |  | T1 |  | 7 | 131.0 | (55.1, 155.6) | 0 |  |  |  |
| Medium (n=182) | Lunch | T0 |  | 129 | 321.2 | (210.1, 457.5) | 133 | 331.7 | (213.8, 437.8) | 0.91 (0.70, 1.18) |
|  |  | T1 |  | 85 | 332.8 | (210.4, 421.3) | 97 | 329.6 | (219.4, 455.6) |  |
|  | Morning snack | T0 |  | 127 | 70.8 | (35.6, 129.6) | 119 | 57.6 | (25.5, 94.6) |  |
|  |  | T1 |  | 82 | 74.8 | (36.7, 121.9) | 76 | 85.3 | (47.0, 129.1) |  |
|  | Afternoon snack | T0 |  | 67 | 156.3 | (64.8, 259.8) | 65 | 53.9 | (21.7, 116.0) |  |
|  |  | T1 |  | 49 | 81.4 | (54.7, 149.9) | 27 | 87.2 | (29.9, 118.1) |  |
| Large (n=142) | Lunch | T0 |  | 76 | 313.4 | (196.4, 407.7) | 100 | 311.4 | (214.5, 459.8) | 0.75 (0.54, 1.05) |
|  |  | T1 |  | 59 | 362.4 | (217.0, 493.0) | 83 | 471.4 | (316.3, 597.4) |  |
|  | Morning snack | T0 |  | 70 | 55.9 | (25.1, 90.5) | 97 | 119.5 | (38.0, 188.0) |  |
|  |  | T1 |  | 56 | 48.0 | (25.6, 65.3) | 69 | 72.3 | (32.0, 140.9) |  |
|  | Afternoon snack | T0 |  | 40 | 50.4 | (35.4, 90.1) | 52 | 137.8 | (59.9, 165.1) |  |
|  |  | T1 |  | 21 | 60.1 | (16.5, 74.5) | 51 | 89.7 | (45.2, 131.8) |  |
| Food provision | |  |  |  |  |  |  |  |  | *Interaction p-value 0.14* |
| Supplied by ECEC provider (n=244) | Lunch | T0 |  | 174 | 316.9 | (212.1, 416.2) | 149 | 297.8 | (191.9, 420.5) | 0.95 (0.77, 1.16) |
|  |  | T1 |  | 120 | 297.0 | (194.9, 420.0) | 124 | 332.1 | (212.8, 448.5) |  |
|  | Morning snack | T0 |  | 219 | 69.3 | (33.1, 129.6) | 200 | 69.3 | (34.0, 133.6) |  |
|  |  | T1 |  | 158 | 61.0 | (34.1, 116.0) | 148 | 70.7 | (35.8, 122.4) |  |
|  | Afternoon snack | T0 |  | 109 | 76.2 | (46.1, 177.5) | 114 | 96.2 | (25.9, 155.6) |  |
|  |  | T1 |  | 79 | 74.5 | (49.6, 132.5) | 74 | 91.4 | (45.2, 130.5) |  |
| Supplied by family e.g. packed lunch (n=128) | Lunch | T0 |  | 74 | 335.0 | (223.9, 485.4) | 103 | 383.1 | (258.8, 453.8) | 0.73 (0.53, 1.02) |
|  |  | T1 |  | 46 | 393.8 | (246.7, 463.3) | 82 | 476.2 | (312.3, 600.0) |  |
|  | Morning snack | T0 |  | 21 | 117.8 | (66.1, 146.3) | 36 | 70.7 | (31.3, 95.0) |  |
|  |  | T1 |  | 1 | 26.4 | (26.4, 26.4) | 18 | 124.0 | (78.5, 193.8) |  |
|  | Afternoon snack | T0 |  | 8 | 79.2 | (19.5, 159.6) | 0 |  |  |  |
|  |  | T1 |  | 1 | 108.9 | (108.9, 108.9) | 1 | 0.0 | (0.0, 0.0) |  |

Abbreviations: ECEC – early childhood education and care; CI – confidence interval; IQR – interquartile range; IMD – index of multiple deprivation.

Note: Data are summarised separately for each eating occasion. Adjusted geometric mean ratio estimated from multilevel model fitting kcal consumed at each eating occasion for each child.

## Table S6: Total Physical Activity subgroup analyses

|  | Data period | Intervention ECEC providers | | | Control ECEC providers | | | Adjusted Mean Difference (95% CI), p-value |
| --- | --- | --- | --- | --- | --- | --- | --- | --- |
|  |  | N | Mean | SD | N | Mean | SD |  |
| Local authority area |  |  |  |  |  |  |  | *Interaction p-value 0.052* |
| Somerset (N=110) | T0 | 61 | 86.9 | 23.9 | 69 | 89.6 | 27.9 | 1.56 (-10.46, 13.58) |
|  | T1 | 49 | 96.8 | 25.1 | 61 | 97.6 | 32.4 |  |
| Swindon (N=35) | T0 | 16 | 91.6 | 28.1 | 33 | 90.7 | 23.7 | 22.47 (-2.08, 47.02) |
|  | T1 | 7 | 110.7 | 29.2 | 28 | 82.6 | 25.5 |  |
| Sandwell (N=19) | T0 | 20 | 83.4 | 31.6 | 28 | 88.4 | 30.8 | -29.14 (-60.64, 2.37) |
|  | T1 | 15 | 76.5 | 27.8 | 4 | 105.0 | 24.3 |  |
| Ayrshire and Arran (N=80) | T0 | 74 | 87.7 | 21.2 | 50 | 83.5 | 27.8 | -9.02 (-22.66, 4.62) |
|  | T1 | 35 | 97.3 | 32.0 | 45 | 102.8 | 23.4 |  |
| Age |  |  |  |  |  |  |  | *Interaction p-value 0.40* |
| <3 years (N=28) | T0 | 0 |  |  | 0 |  |  | 11.29 (-9.75, 32.33) |
|  | T1 | 12 | 80.0 | 27.7 | 16 | 70.1 | 22.8 |  |
| 3-4 years (N=117) | T0 | 14 | 74.5 | 22.6 | 16 | 65.9 | 26.0 | -3.76 (-14.94, 7.43) |
|  | T1 | 49 | 92.7 | 22.8 | 68 | 96.3 | 27.2 |  |
| >4 years (N=97) | T0 | 22 | 76.9 | 20.0 | 20 | 73.0 | 24.0 | -3.31 (-15.35, 8.74) |
|  | T1 | 43 | 102.3 | 34.2 | 54 | 104.4 | 28.1 |  |
| Sex |  |  |  |  |  |  |  | *Interaction p-value 0.43* |
| Male (N=113) | T0 | 76 | 93.2 | 25.5 | 97 | 90.4 | 27.7 | -3.49 (-15.01, 8.02) |
|  | T1 | 43 | 99.4 | 33.9 | 70 | 103.2 | 29.9 |  |
| Female (N=131) | T0 | 94 | 82.2 | 21.8 | 82 | 85.2 | 27.4 | 1.93 (-8.84, 12.71) |
|  | T1 | 63 | 92.0 | 24.9 | 68 | 89.5 | 26.1 |  |
| Child deprivation status |  |  |  |  |  |  |  | *Interaction p-value 0.19* |
| High deprivation (N=45) | T0 | 41 | 85.2 | 26.7 | 39 | 91.3 | 32.5 | 2.11 (-17.04, 21.27) |
|  | T1 | 31 | 100.3 | 26.7 | 14 | 98.6 | 25.8 |  |
| Moderate deprivation (N=181) | T0 | 113 | 87.9 | 21.8 | 124 | 84.8 | 25.6 | -4.59 (-14.20, 5.01) |
|  | T1 | 74 | 92.1 | 29.3 | 107 | 95.8 | 29.9 |  |
| Low deprivation (N=18) | T0 | 17 | 88.0 | 32.3 | 17 | 102.9 | 24.4 | 47.12 (-8.93, 103.17) |
|  | T1 | 1 | 145.6 | - | 17 | 98.4 | 25.3 |  |
| ECEC provider size |  |  |  |  |  |  |  | *Interaction p-value 0.66* |
| Small (<30) (N=43) | T0 | 35 | 90.1 | 16.2 | 29 | 96.8 | 21.4 | -3.25 (-22.31, 15.82) |
|  | T1 | 21 | 89.4 | 31.7 | 22 | 96.5 | 30.2 |  |
| Medium (30-60) (N=107) | T0 | 81 | 84.5 | 27.3 | 97 | 86.8 | 29.1 | 2.67 (-10.75, 16.08) |
|  | T1 | 47 | 95.4 | 29.3 | 60 | 92.7 | 26.8 |  |
| Large (>60) (N=91) | T0 | 45 | 89.6 | 21.1 | 54 | 85.1 | 27.1 | -6.61 (-21.72, 8.49) |
|  | T1 | 35 | 97.0 | 28.3 | 56 | 100.4 | 30.4 |  |

Abbreviations: ECEC – early childhood education and care; CI – confidence interval; SD – standard deviation.

## Table S7: Sensitivity analyses

| Sensitivity analyses | | Adjusted effect estimate (95% CI) | p-value |
| --- | --- | --- | --- |
| Total Physical Activity | | *Adjusted mean difference (95% CI)* |  |
|  | Primary analysis | -2·13 (-10·96, 6·70) | 0.64 |
|  | Based on data recorded over the full day | -2.06 (-12.83, 8.71) |  |
|  | Per-protocol | -4.20 (-15.39, 7.00) |  |
|  | Compliance level 1 (a) | -3.12 (-12.36, 6.11) |  |
|  | Compliance level 2 (b) | 3.68 (-6.38, 13.75) |  |
|  | Additional covariates (c) | 0.72 (-8.90, 10.34) |  |
|  | Adjusting for seasonality (d) | -1.69 (-10.71, 7.34) |  |
| Energy consumed across all eating occasions | | *Adjusted geometric mean ratio (95% CI)* |  |
|  | Primary analysis | 0·86 (0·72, 1·03) | 0·094 |
|  | Per protocol | 0.87 (0.73, 1.05) |  |
|  | Compliance level 1 (a) | 0.88 (0.74, 1.06) |  |
|  | Compliance level 2 (b) | 0.88 (0.67, 1.17) |  |
|  | Excluding outliers | 0.83 (0.71, 0.98) |  |
|  | Additional covariates (c) | 0.87 (0.72, 1.04) |  |
|  | Adjusting for seasonality (d) | 0.87 (0.74, 1.04) |  |

Abbreviations: ECEC – early childhood education and care; CI – confidence interval.

a Four ECEC providers (17 enrolled children) in intervention group excluded from analyses.

b Thirteen ECEC providers (84 enrolled children) in intervention group excluded from analyses.

c adjusted for child age, standardised body mass index (zBMI) and sex at T1 in addition to the covariate adjustment in the primary analysis. NOTE: baseline nutrition was averaged across all children within the ECEC setting and not adjusted for the age, zBMI or sex of children at this time point.

d adjustment for hours of daylight on date of T1 nutrition data and average hours of daylight of all children within the ECEC setting at T0 in addition to the covariate adjustment in the primary analysis. This is to try and adjust the baseline average for seasonality, as it was demonstrated that baseline data collection spanned a wider range of seasons and took place over a year, compared to T1 data collection that was predominantly over the Winter.

## Figure S5: Results from post-hoc nutrition analyses

*Post-hoc nutrition analyses*

A post-hoc subgroup analysis by country (interaction p-value 0·042) demonstrated an intervention effect on kcal consumed across all ECEC providers in England, but not in Scotland (appendix p10-14). Two per cent of children in Scotland had packed lunches. In contrast, half the children in English ECEC settings had lunchboxes provided by parents (51.5% at baseline and 48% at follow-up) which was more than anticipated from the feasibility study, where only a small number had packed lunches. At baseline the energy of lunches served were higher than recommended, with lunchboxes 63% higher (median (IQR) 586·6kcal (454·6, 715·0)) and ECEC providers 14% higher (median 408·0 kcal (309·6, 519·8)). The intervention effect did not differ based on type of food provision (ECEC-provided vs. parent-provided) in a post-hoc subgroup analysis (interaction p=0·14) (see appendix p18). Given the intervention effect demonstrated in kcal consumed and served within lunches, these subgroup analyses were repeated on each of the nutrition outcomes restricted to lunches only. Consumed and served kcal were similar between the two arms for ECEC provided meals, but lower in the intervention arm for lunchboxes, however the intervention effect was not different by meal provision (interaction p=0·069 and p=0·082 respectively). There was evidence for a differential intervention effect for % kcal consumed from non-core foods by type of food provision (interaction p=0·027, appendix p18); % kcal from non-core foods was lower with the intervention in ECEC provided food with an aMD of -11·5% (95%CI -22·6, -0·4); p=0·042), however there was no evidence for an intervention effect on % kcal for non-core food in lunchboxes (aMD: 7·9% (95%CI -6·1, 22·0); p=0·27).

## Table S8: kcal consumed, kcal served and % kcal from non-core food summarised by type of food provision for lunches (including England and Scotland)

|  | Food provision | Data period | Intervention ECEC providers | | | Control ECEC providers | | | Adjusted Mean Difference (95% CI), p-value  *model excluding interaction* | Adjusted Mean Difference (95% CI), p-value  *subgroup analyses* |
| --- | --- | --- | --- | --- | --- | --- | --- | --- | --- | --- |
|  |  |  | N | Median | IQR | N | Median | IQR |  |  |
| Kcal consumed | | |  |  |  |  |  |  | -67.7 (-118.6, -16.7), p=0.009 | Interaction p-value 0.069 |
|  | Supplied by ECEC provider | T0 | 174 | 316.9 | (212.1, 416.2) | 149 | 297.8 | (191.9, 420.5) |  |  |
|  |  | T1 | 120 | 297.0 | (194.9, 420.0) | 124 | 332.1 | (212.8, 448.5) |  | -21.3 (-82.4, 39.8) |
|  | Packed lunches | T0 | 74 | 335.0 | (223.9, 485.4) | 103 | 383.1 | (258.8, 453.8) |  |  |
|  |  | T1 | 46 | 393.8 | (246.7, 463.3) | 82 | 476.2 | (312.3, 600.0) |  | -113.9 (-194.7, -33.2) |
| Kcal served | | |  |  |  |  |  |  | -69.1 (-116, -22.2), p=0.004 | Interaction p-value 0.082 |
|  | Supplied by ECEC provider | T0 | 174 | 418.6 | (345.0, 533.6) | 149 | 388.6 | (280.4, 513.9) |  |  |
|  |  | T1 | 120 | 408.0 | (296.9, 536.3) | 124 | 408.0 | (329.1, 510.4) |  | -15.4 (-74.4, 43.6) |
|  | Packed lunches | T0 | 74 | 498.1 | (401.5, 641.0) | 103 | 524.5 | (430.1, 671.3) |  |  |
|  |  | T1 | 46 | 508.0 | (419.4, 645.6) | 82 | 647.5 | (475.8, 737.8) |  | -102.7 (-180.9, -24.5) |
| % non-core | |  |  |  |  |  |  |  | -5.4 (-14.7, 4.0), p=0.26 | Interaction p-value 0.027 |
|  | Supplied by ECEC provider | T0 | 174 | 30.9 | (3.5, 50.1) | 148 | 33.9 | (0, 62.4) |  |  |
|  |  | T1 | 119 | 14.8 | (0, 47.4) | 122 | 35.0 | (6.8, 59.3) |  | -11.5 (-22.6, -0.4) |
|  | Packed lunches | T0 | 74 | 50.4 | (28.4, 65.2) | 102 | 46.6 | (34.0, 64.4) |  |  |
|  |  | T1 | 46 | 51 | (32.5, 70.1) | 82 | 44.1 | (29.1, 59.9) |  | 7.9 (-6.1, 22.0) |

Abbreviations: ECEC – early childhood education and care; CI – confidence interval; IQR – interquartile range.

## Table S9: ECEC provider mediator questionnaires[9]

|  | | Intervention ECEC providers | | | | | | Control ECEC providers | | | | | |
| --- | --- | --- | --- | --- | --- | --- | --- | --- | --- | --- | --- | --- | --- |
|  | | T0 | | | T1 | | | T0 | | | T1 | | |
|  | | N | Median | IQR | N | Median | IQR | N | Median | IQR | N | Median | IQR |
| **PA, play and sedentary time** | |  |  |  |  |  |  |  |  |  |  |  |  |
|  | Knowledge | 111 | 2.8 | (2.2, 3.5) | 43 | 3.2 | (2.2, 3.2) | 124 | 2.6 | (2.2, 3.5) | 58 | 2.6 | (2.2, 3.2) |
|  | Knowledge (%) | 111 | 68.8 | (56.2, 87.5) | 43 | 81.2 | (56.2, 81.2) | 124 | 65.6 | (56.2, 87.5) | 58 | 65.6 | (56.2, 81.2) |
|  | Motivation (max 5) | 115 | 4.8 | (4.1, 5.0) | 43 | 4.9 | (4.4, 5.0) | 127 | 4.6 | (4.1, 5.0) | 61 | 4.7 | (4.2, 5.0) |
|  | Self-efficacy (max 5) | 115 | 4.6 | (4.1, 4.9) | 42 | 4.9 | (4.6, 5.0) | 127 | 4.5 | (3.9, 4.9) | 59 | 4.6 | (4.2, 4.9) |
| **Nutrition** | |  |  |  |  |  |  |  |  |  |  |  |  |
|  | Knowledge | 107 | 5.3 | (4.8, 5.8) | 39 | 5.5 | (5.1, 5.6) | 121 | 5.2 | (4.6, 5.6) | 62 | 5.3 | (4.7, 5.7) |
|  | Knowledge (%) | 107 | 88.3 | (80.0, 95.8) | 39 | 91.7 | (84.4, 93.3) | 121 | 86.9 | (76.7, 93.3) | 62 | 89.2 | (77.5, 94.4) |
|  | Motivation (max 5) | 116 | 4.5 | (3.9, 5.0) | 44 | 5.0 | (4.2, 5.0) | 125 | 4.4 | (3.8, 4.8) | 60 | 4.6 | (4.1, 5.0) |
|  | Self-efficacy (max 5) | 116 | 4.4 | (3.7, 4.7) | 44 | 4.6 | (4.2, 5.0) | 127 | 4.2 | (3.6, 4.6) | 60 | 4.5 | (3.8, 4.7) |

Abbreviations: ECEC – early childhood education and care; IQR – interquartile range; PA – physical activity.

PA knowledge score calculated as a total score out of 4, converted to a % (also presented);

PA motivation score calculated as an average score across 10 questions, each scored from 1 (lowest) to 5 (highest);

PA self-efficacy score calculated as an average score across 10 questions, each scored from 1 (lowest) to 5 (highest);

Nutrition knowledge score calculated as a total score out of 6, converted to a % (also presented);

Nutrition motivation score calculated as an average score across 9 questions, each question scored from 1 (lowest) to 5 (highest);

Nutrition self-efficacy score calculated as an average score across 9 questions, each question scored from 1 (lowest) to 5 (highest).

## Table S10: Parent Mediator questionnaires[9]

|  | | Intervention ECEC providers | | | | | | Control ECEC providers | | | | | |
| --- | --- | --- | --- | --- | --- | --- | --- | --- | --- | --- | --- | --- | --- |
|  | | T0 | | | T1 | | | T0 | | | T1 | | |
|  | | N | Median | IQR | N | Median | IQR | N | Median | IQR | N | Median | IQR |
| **PA, play and sedentary time** | |  |  |  |  |  |  |  |  |  |  |  |  |
|  | Knowledge | 159 | 2.8 | (2.2, 3.0) | 103 | 2.5 | (2.2, 3.0) | 188 | 2.6 | (2.2, 3.0) | 139 | 2.5 | (2.2, 3.0) |
|  | Knowledge (%) | 159 | 68.8 | (56.2, 75.0) | 103 | 62.5 | (56.2, 75.0) | 188 | 65.6 | (56.2, 75.0) | 139 | 62.5 | (56.2, 75.0) |
|  | Motivation (max 5) | 165 | 4.3 | (3.8, 5.0) | 109 | 4.4 | (3.7, 4.9) | 193 | 4.4 | (4.0, 4.8) | 145 | 4.4 | (4.0, 4.9) |
|  | Self-efficacy (max 5) | 165 | 4.5 | (3.9, 4.8) | 109 | 4.4 | (3.9, 4.8) | 194 | 4.5 | (4.1, 4.8) | 146 | 4.5 | (4.0, 4.8) |
| **Nutrition** | |  |  |  |  |  |  |  |  |  |  |  |  |
|  | Knowledge | 161 | 5.2 | (4.5, 5.7) | 107 | 5.2 | (4.8, 5.5) | 193 | 5.2 | (4.7, 5.6) | 144 | 5.2 | (4.8, 5.7) |
|  | Knowledge (%) | 161 | 85.8 | (75.3, 94.4) | 107 | 85.8 | (79.2, 91.7) | 193 | 86.9 | (77.8, 93.3) | 144 | 87.5 | (80.3, 94.4) |
|  | Motivation (max 5) | 165 | 4.4 | (4.0, 4.9) | 108 | 4.4 | (3.8, 4.8) | 194 | 4.3 | (3.9, 4.7) | 145 | 4.5 | (4.0, 4.8) |
|  | Self-efficacy (max 5) | 164 | 4.6 | (4.1, 4.8) | 107 | 4.6 | (4.0, 4.9) | 193 | 4.4 | (3.9, 4.8) | 145 | 4.6 | (4.1, 4.9) |

Abbreviations: ECEC – early childhood education care; IQR – interquartile range; PA – physical activity.

PA knowledge score calculated as a total score out of 4, converted to a % (also presented);

PA motivation score calculated as an average score across 14 questions, each scored from 1 (lowest) to 5 (highest);

PA self-efficacy score calculated as an average score across 14 questions, each scored from 1 (lowest) to 5 (highest);

Nutrition knowledge score calculated as a total score out of 6, converted to a % (also presented);

Nutrition motivation score calculated as an average score across 11 questions, each question scored from 1 (lowest) to 5 (highest);

Nutrition self-efficacy score calculated as an average score across 11 questions, each question scored from 1 (lowest) to 5 (highest).

## Table S11: Review and Reflect Tool


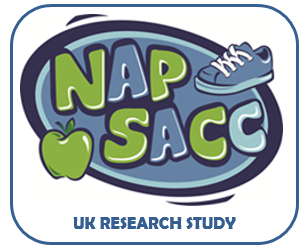


**Nutrition and Physical Activity Self-Assessment for Childcare**

**Review & Reflect**

NAP SACC UK is based on a set of best practices that stem from the latest research and guidelines in the field. After completing the Review & Reflect, you will be able to see your nursery’s strengths and areas for improvement and use this information to plan healthy changes.

**Before you begin:**

✓ Gather menus, staff manuals, parent handbooks, and other documents that state your policies and guidelines about child nutrition, physical activity, outdoor play & learning, and screen time.

✓ Involve staff members who are familiar with day-to-day practices.

**As you reflect:**

✓ The question and answers are based on full day care including breakfast, lunch, tea and snacks. If your nursery only provides half-day care you may need to estimate what your answer would be.

✓ Definitions of key words are marked by asterisks (*).

✓ Answer each question as best you can. If none of the answer choices seem quite right, just pick the closest fit. If a question does not apply to your nursery, move to the next question.

**Using your review and reflect form:**

✓ Once you have completed this form your NAP SACC UK partner will arrange a time to meet with you. You will review your answers together, find areas for improvement and set goals to help make your nursery a healthier place.

✓ Your staff will be having two NAP SACC UK training sessions; one on nutrition and one on physical activity. This will be an opportunity for your staff to meet together to plan actions against the goals you have set.

| Nursery Name |  |
| --- | --- |
| Name of member of staff completing Review and Reflect |  |
| Date completed |  |
| Date discussed with  NAP SACC UK Partner |  |

*NAP SACC is* *Copyright* *of University of North Carolina at Chapel Hill*

*Center for Health Promotion and Disease Prevention (HPDP)*

*.*

*NAP SACC UK was adapted for use in the UK by the University of Bristol under funding from NIHR.*

 Child Nutrition

| Office use only: | |
| --- | --- |
| Nursery ID |  |
| Nursery Staff ID |  |
| Partner ID |  |

For this self-assessment, child nutrition topics include foods and drinks provided to children, as well as the environment and staff practices during meal times. Unless otherwise noted, all questions in this section relate to your nursery’s practices for both toddlers and preschool children.

Foods Provided

| 1. | Guidelines exist to help nurseries provide balanced menus for the children in their care.  In England the guidelines are available from the Government (Public Health England) and are supported by the Action for Children ‘Eat Better, Start Better practical guide’.* In Scotland the guidelines are ‘Setting the Table’^. | | | |
| --- | --- | --- | --- | --- |
|  | **□** We are not aware of the guidelines | **□** We are aware of the guidelines but *rarely* refer to them | **□** We are aware of the guidelines and *often* refer to them | **□** We are fully aware of the guidelines and follow them whenever possible |
|  | * In England the Government guidance is:  - [Example menus for early years settings in England: part 1 (publishing.service.gov.uk)](https://assets.publishing.service.gov.uk/government/uploads/system/uploads/attachment_data/file/658870/Early_years_menus_part_1_guidance.pdf)  And supported by:  - ‘Eat Better Start Better a practical guide’ published by Action for Children in 2017 <https://foundationyears.org.uk/wp-content/uploads/2017/11/Eat-Better-Start-Better1.pdf>  ^ In Scotland the guidance is:  [Setting the table (healthscotland.com)](http://www.healthscotland.com/uploads/documents/30341-Setting%20the%20Table.pdf) | | | |

| 2. | This question is for nurseries who provide the option of breakfast. If your nursery does not offer breakfast, please move on:  Breakfast is an important meal for young children. We ensure that we: | | | |
| --- | --- | --- | --- | --- |
|  | Tick all that apply:   - Liaise with parents to ensure children always eat breakfast, whether at home or in the nursery - Provide a portion of fruit or vegetable at breakfast each day - Choose cereals with low or medium sugar content and avoid cereals that are high in sugar such as sugar coated or chocolate flavoured cereals - Provide a portion of starchy food* as part of breakfast each day - Provide at least 3 different varieties of starchy food* across breakfasts each week | | | |
|  | **□** 0-1 ticks | **□** 2-3 ticks | **□** 4 ticks | **□** 5 ticks |
|  | *****Starchy foods could include types of cereals, porridge, bread, crumpets, English style muffins, rice cakes, breadsticks, low salt crackers | | | |

| 3. | At all main meals*: | | | |
| --- | --- | --- | --- | --- |
|  | **□** A portion^✝^ of fruit or veg is sometimes provided | **□** A portion^✝^ of fruit or veg is *usually* provided | **□** At least 1 portion^✝^ of fruit *or* 1 portion of veg is *always* served | **□** At least 1 portion^✝^ of fruit *and* 1 portion of veg are *always* served |
|  | * for main meals include lunch and tea, do not include breakfast  ^✝^ Portion sizes for children are outlined on page 19 of the ‘Eat Better Start Better’ guidelines. Fruit and vegetables can be included as part of a composite meal e.g. mushrooms in lasagne. | | | |

Please continue to question 4 on the next page

| 4. | Over the course of a week, snacks are provided as follows: | | | |
| --- | --- | --- | --- | --- |
|  | Tick all that apply:   - Fruit and vegetables are provided as part of some snacks - Dried fruit is not provided as part of snacks - Starchy food is provided as part of at least 1 snack per day - At least 3 different types of starchy food* are used in snacks across a whole week - Meat, fish and other non-dairy sources of protein^✝^ are included as part of a snack once or twice per week - Dairy foods^^^ are provided as part of a snack once or twice per week | | | |
|  | **□** 1-2 ticks | **□** 3-4 ticks | **□** 5 ticks | **□** 6 ticks |
|  | *****Starchy foods could include types of bread, cereals, crumpets, English style muffins, rice cakes, breadsticks, low salt crackers  ^✝^Other non-dairy sources of protein could include pulses (beans, chickpeas, lentils)  ^^^Dairy foods could include milk, cheese, yoghurt, fromage frais | | | |

| 5. | When we serve vegetables:* | | | |
| --- | --- | --- | --- | --- |
|  | **□** We offer 1-3 different *types* of vegetables across a week | **□** We offer 4-5 different *types* of vegetable across the week | **□** We offer 6-7 different *types* of vegetable across the week | **□** We offer 8 or more different *types* of vegetable across the week |
|  | * For this question, potato is not included as a vegetable, but sweet potato is included. Vegetables can be included as part of a composite meal e.g. mushrooms in lasagne. | | | |

| 6. | Our nursery offers processed potatoes:* | | | |
| --- | --- | --- | --- | --- |
|  | **□** 3 times per week or more | **□** 2 times per week | **□** Once per week | **□** Less than once per week or never |
|  | * Processed potatoes are those that are pre-fried and sold frozen, then prepared in the oven. They include frozen chips, smiley faces, waffles, wedges, hash browns, croquettes etc. | | | |

| 7. | Our nursery offers processed meat products or processed fish products:* | | | |
| --- | --- | --- | --- | --- |
|  | **□** 3 times per week or more | **□** 2 times per week | **□** Once per week | **□** Less than once per week or never |
|  | * Processed meat or fish products include breaded and frozen chicken nuggets and fish fingers, and processed meat products such as sausages, bacon, salami/pepperoni; homemade fish fingers or chicken nuggets are not included. | | | |

| 8. | Our nursery provides meat-free meals containing meat alternatives:* | | | |
| --- | --- | --- | --- | --- |
|  | **□** Never, all meals contain meat | **□** Less than once per week | **□** For at least 1 meal per week | **□** For at least 1 lunch and 1 tea each week |
|  | * Meat alternatives are sources of protein that do not contain meat, such as pulses (e.g. beans, chickpeas and lentils), eggs, Quorn pieces or Tofu. | | | |

Please continue to question 9 on the next page.

| 9. | Over the course of a week, meat or meat alternatives are served from the following categories to ensure variety*: | | | |
| --- | --- | --- | --- | --- |
|  | Tick all that apply:   - Poultry - Fish - Red meat - Meat alternatives^^^ - Oily fish^✝^ | | | |
|  | **□** 1-2 tick | **□** 3 ticks | **□** 4 ticks | **□** 5 ticks |
|  | *****These can be served as part of lunch or tea  ^^^Meat alternatives include pulses (beans, chickpeas, lentils), eggs, or meat-free alternatives such as Quorn pieces or Tofu.  ^✝^Oily fish should be included weekly, but should **not** be served more than twice per week. Oily fish includes salmon, sardines or pilchards. Oily fish includes tinned salmon, but does not include tinned tuna. | | | |

| 10. | The starchy foods we serve:* | | | |
| --- | --- | --- | --- | --- |
|  | **□** Include 2 or fewer types over the course of the *week* | **□** Include at least 3 types over the course of the *week* | **□** Include at least 3 types over the course of the *day* | **□** Include at least 3 types over the course of the *day* incorporating both white *and* wholegrain products^✝^ |
|  | * Starchy foods include bread, pasta, rice, noodles, cous cous and breakfast cereals. ^✝^Wholegrain starchy foods could include brown bread, oats and wholegrain breakfast cereals. | | | |

| 11. | Children need 3 portions of milk or dairy food* every day. Two of these are likely to be served at home each day. We ensure we: | | | |
| --- | --- | --- | --- | --- |
|  | Tick all that apply:   - Provide a portion of milk or dairy food per day as a drink, snack or part of a meal - Choose yoghurts or fromage frais that have a lower sugar content^✝^and serve them as part of a main meal - Only serve plain full-fat or semi skimmed milk, not flavoured milk - Only serve ice-cream with fruit desserts as part of a meal and no more than once per week | | | |
|  | **□** Dairy is not provided daily | **□** 1-2 ticks | **□** 3 ticks | **□** 4 ticks |
|  | *A portion of dairy could include 100-150ml of milk (half a cup), 20-30g of cheese, small pot (60g) or half a large individual pot (half of 125-140g) of yoghurt/fromage frais, 1-2 tablespoons of yoghurt from a multi-serving pot, 3-4 tablespoons of a milk-based pudding (custard, rice pudding or semolina)  ^✝^A lower sugar content would be no higher than 15g sugar per 100g yoghurt. | | | |

Please continue to question 12 on the next page

| 12. | Our nursery is aware that it is important to limit children’s salt intake, and do so in the following ways: | | | |
| --- | --- | --- | --- | --- |
|  | Tick all that apply:   - We do not add salt when cooking - We do not provide salt on the tables for the children to add to their food - We limit the use of ready-made sauces, soups, stocks or gravy granules - We limit the use of sauces on the table such as ketchup - We never serve salty snacks such as crisps | | | |
|  | **□** 0-1 ticks | **□** 2-3 ticks | **□** 4 ticks | **□** 5 ticks |

| 13. | Our nursery offers high-sugar or high-fat food as *snacks*:* | | | |
| --- | --- | --- | --- | --- |
|  | **□** Once per day or more | **□** 3-4 times per week | **□** 1-2 times per week | **□** Less than once per week or never |
|  | * High-sugar or high-fat snacks include biscuits, cakes, doughnuts, American style muffins, chocolate, ice cream | | | |

| 14. | Over the course of a week, puddings are served from the following categories: | | | |
| --- | --- | --- | --- | --- |
|  | Tick all that apply:   - Hot fruit-based desserts such as crumbles or baked apples - Milk-based desserts such as semolina, rice pudding and custard - Yoghurt or fromage frais - Cakes and biscuits containing fruit, such as carrot cake or fruit flapjack - Cold desserts such as fruit salad or a piece of fruit | | | |
|  | **□** 0 ticks | **□** 1-2 ticks | **□** 3-4 ticks | **□** 5 ticks |

| 15. | Cakes and/or other sweet or high fat foods are used to celebrate events*: | | | |
| --- | --- | --- | --- | --- |
|  | **□** Approximately weekly or more frequently | **□** Approximately monthly | **□** Approximately every other month | **□** 2-3 times per year, for major celebrations or religious festivals only, or never |
|  | * Events could include holidays, religious festivals, birthdays or other celebrations | | | |

Beverages Provided

| 16. | Drinking water is available:* | | | |
| --- | --- | --- | --- | --- |
|  | **□** Only when children ask | **□** Only when children ask and during water breaks | **□** Only indoors, where it is always visible and freely available* | **□** Indoors and outdoors, where it is always visible and freely available* |
|  | *Water that is “freely available” is always available to children but may or may not be ‘help yourself’. Water may be available from water bottles/beakers, jugs, portable or fixed water coolers, or water fountains. | | | |

Please continue to question 17 on the next page

| 17. | Our nursery offers children fruit juice*: | | | |
| --- | --- | --- | --- | --- |
|  | **□** More than once per day without dilution | **□** Once per day or less without dilution | **□** Once per day, and this is diluted down with at least 50% water | **□** Less than once per day, only at meal times, and this is diluted with at least 50% water and served in an open cup |
|  | * Fruit juice means 100% or pure fruit juice. This may either be labelled as ‘fresh’, ’not from concentrate’ or ‘from concentrate’. This does not include ‘fruit juice *drinks*’ which generally only contain a small amount of fruit juice and a high level of added sugar. | | | |

| 18. | Our nursery offers sugary drinks:* | | | |
| --- | --- | --- | --- | --- |
|  | **□** Once per month or more | **□** Once every few months | **□** 1-2 times per year | **□** Never |
|  | *Sugary drinks include squash, energy drinks and sugar-sweetened fizzy drinks. | | | |

Questions 19-21 are intentionally missing for this study

Feeding Environment and Practices

| 22. | Meals and snacks are served to preschool children in the following way:* | | | |
| --- | --- | --- | --- | --- |
|  | **□** Meals and snacks come pre-plated with set portions of each food | **□** Staff portion out servings to children | **□** Children serve some food themselves, while other foods are pre-plated or served by staff | **□** Children* choose and serve most foods themselves with adult supervision to help portion size |
|  | * This refers to preschool children who are developmentally ready to choose and serve foods themselves. | | | |

| 23. | Television or videos are on during meal or snack times: | | | |
| --- | --- | --- | --- | --- |
|  | **□** Always | **□** Often | **□** Sometimes | **□** Never |

| 24. | During meal or snack times, staff eat and drink the same food and drink as children: | | | |
| --- | --- | --- | --- | --- |
|  | **□** Rarely or never | **□** Sometimes | **□** Often | **□** Always |

| 25. | Staff eat or drink unhealthy foods or beverages in front of children: | | | |
| --- | --- | --- | --- | --- |
|  | **□** Daily | **□** Often | **□** Sometimes | **□** Rarely or never |

| 26. | Staff enthusiastically role model* eating healthy foods served at meal and snack times: | | | |
| --- | --- | --- | --- | --- |
|  | **□** Rarely or never | **□** Sometimes | **□** Often | **□** Every meal and snack time |
|  | *Enthusiastic role modelling is when staff eat healthy foods in front of children and show how much they enjoy them. For example, a staff member might say, “Mmmm, these peas taste yummy!” | | | |

Please continue to question 27 on the next page

| 27. | Staff praise children for trying new or less-preferred foods: | | | |
| --- | --- | --- | --- | --- |
|  | **□** Rarely or never | **□** Sometimes | **□** Often | **□** Always |

| 28. | When children eat less than half of a meal or snack, staff ask them if they are full before removing their plates: | | | |
| --- | --- | --- | --- | --- |
|  | **□** Rarely or never | **□** Sometimes | **□** Often | **□** Always |

| 29. | When children request seconds, staff ask them if they are still hungry before serving more food: | | | |
| --- | --- | --- | --- | --- |
|  | **□** Rarely or never | **□** Sometimes | **□** Often | **□** Always |

| 30. | Children are required to finish everything on their plate: | | | |
| --- | --- | --- | --- | --- |
|  | **□** Every meal and snack time | **□** Usually | **□** Sometimes | **□** Rarely or never |

| 31. | During physically active playtime and in hot weather, staff remind children to drink water: | | | |
| --- | --- | --- | --- | --- |
|  | **□** Rarely or never | **□** Sometimes | **□** Often | **□** At least once per play period |

| 32. | Children eat snacks and meals: | | | |
| --- | --- | --- | --- | --- |
|  | **□** In their usual room with no change; toys, equipment etc are left out during meal times | **□** In their usual room; toys, equipment etc are sometimes tidied away, or some differentiation* is *sometimes* used | **□** In their usual room, but snack and meal times are *always* clearly differentiated from learning/ playtime* | **□** In a separate room^✝^ or separate area within the room^^^ to where they learn/play |
|  | *To differentiate between meal times and learning/play times, a song could be used to mark the change to eating time, toys and equipment could be tidied away and cloths could be put on tables  ^✝^The separate room could be a side room or a dining room  ^^^ This would be a separate area within the room that is used for mealtimes only | | | |

Menus and Variety

| 33. | The length of our nursery’s menu cycle* is: | | | |
| --- | --- | --- | --- | --- |
|  | **□** 1 week or shorter | **□** 2 weeks | **□** 3 weeks or longer without seasonal change | **□** 3 weeks or longer^✝^, with seasonal change |
|  | *The length of the menu cycle is the length of time that it takes for the menu to repeat.  ^✝^Ideally the menu cycle would start on a different day each cycle, to ensure children who are only in care part time receive a full variety of meals. | | | |

Education and Professional Development

| 34. | Staff talk with children informally about healthy eating: | | | |
| --- | --- | --- | --- | --- |
|  | **□** Rarely or never | **□** Sometimes | **□** Often | **□** Each time they see an opportunity |

Please continue to question 35 on the next page.

| 35. | Staff incorporate healthy eating learning into their children’s formal daily activities, play and learning experiences: | | | |
| --- | --- | --- | --- | --- |
|  | **□** Once per month or less | **□** 2-3 times per month | **□** Once per week or more | **□** More than once per week |
|  | *Healthy eating learning can include circle time lessons, story time, cooking activities and gardening activities. | | | |

| 36. | Cooking and other activities teaching skills around handling and preparing healthy foods* are included in learning experiences: | | | |
| --- | --- | --- | --- | --- |
|  | **□** Never | **□** Once per month or less | **□** 2-3 times per month | **□** Once per week or more |
|  | *Activities around handling and preparing healthy foods could include learning to safely chop up fruit and veg | | | |

| 37. | Staff receive professional development* on child nutrition: | | | |
| --- | --- | --- | --- | --- |
|  | **□** Never | **□** Less than once per year | **□** Once per year | **□** 2 times per year or more |
|  | *For this question, professional development on child nutrition does not include training on food safety. Professional development can include talking in person or online training for contact hours or continuing professional development credits. It can also include information presented at staff meetings. Training should be from a reputable source (e.g. NHS, Local Authority or National Organisation). | | | |

| 38. | Professional development for current staff on child nutrition has included the following topics: | | | |
| --- | --- | --- | --- | --- |
|  | Tick all that apply:   - Food and drink recommendations for children - Serving sizes for children - Importance of variety in the children’s diet - Creating healthy mealtime environments* - Using positive feeding practices to support children to eat well^✝^ - Supporting staff to communicate with families about child nutrition - Your nursery’s policies on child nutrition | | | |
|  | **□** 0 ticks | **□** 1-3 ticks | **□** 4-5 ticks | **□** 6-7 ticks |
|  | *****In a healthy mealtime environment, children can choose what to eat from the foods offered, television and videos are turned off, and staff sit with children and enthusiastically role model eating healthy foods.  ^✝^Positive feeding practices include praising children for trying new foods, asking children about hunger/fullness before taking their plates away or serving seconds, and avoiding the use of food to calm children or to encourage appropriate behaviour. | | | |

| 39. | Families are offered information and support* on child nutrition: | | | |
| --- | --- | --- | --- | --- |
|  | **□** Never | **□** Less than once per year | **□** Once per year | **□** 2 times per year or more |
|  | *****Information and support can be offered through in-person educational sessions, brochures, leaflets, tip sheets, your nursery’s newsletter, website, or bulletin boards, through signposting to initiatives such as Change4Life, Healthy Start, or local food co-operatives or cooking groups, or through opportunistic conversations with parents about their child’s eating habits and intake. | | | |

| 40. | Information or support for families on child nutrition includes the following topics: | | | |
| --- | --- | --- | --- | --- |
|  | Tick all that apply:   - Food and beverage recommendations for children - Serving sizes for children - Importance of variety in the children’s diet - Creating healthy mealtime environments - Using positive feeding practices - Your nursery’s policies on child nutrition | | | |
|  | **□** 0 ticks | **□** 1-2 ticks | **□** 3-4 ticks | **□** 5-6 ticks |

Policy

| 41. | Our written policy* on child nutrition includes the following topics: | | | |
| --- | --- | --- | --- | --- |
|  | Tick all that apply:   - Foods provided to children - Beverages provided to children - Creating healthy mealtime environments - Teaching practices to encourage healthy eating - Not offering food to calm children or encourage appropriate behaviour - Planned and informal nutrition education for children - Professional development on child nutrition - Education for families on child nutrition - Guidelines for food offered for seasonal/religious celebrations, birthdays etc - Provision of special diets | | | |
|  | **□** No written policy or policy does not include these topics | **□** 1-5 ticks | **□** 6-8 ticks | **□** 9-10 ticks |
|  | *****A written policy can include any written guidance about your nursery’s operations or expectations for staff, children, and families. Policies can be included in parent handbooks, staff manuals, and other documents. Ideally staff, parents and children are involved in writing the policy. | | | |

 **Child Nutrition Health**

**Lunchboxes**

The questions in this section are only relevant if your nursery has children bringing in food or drink for lunchtime. If your nursery does not have lunchboxes brought from home, please move on.

For the following statements think about the typical food which is provided in lunchboxes.

| 42. | On a typical day, lunchboxes are ‘balanced’ as they contain food from each of the 4 foods groups* and limit foods and drinks high in saturated fat, sugar and salt: | | | |
| --- | --- | --- | --- | --- |
|  | **□** Few (or no) lunchboxes are balanced | **□** Less than half of lunchboxes balanced | **□** Half or more lunchboxes are balanced | **□** Almost all (or all) lunchboxes are balanced |
|  | *** A balanced lunch should include the following:   - a portion of fruit and vegetables - a portion of starchy foods (potatoes, bread, rice, pasta and other carbohydrates), ideally wholegrain or unprocessed - a portion of protein (meat, fish, pulses, eggs, and other proteins) - a portion of dairy foods or alternatives (milk, cheese, yoghurt, fromage frais) | | | |

| 43. | On a typical day, lunchboxes have fruit provided as follows: | | | |
| --- | --- | --- | --- | --- |
|  | **□** Few (or no) lunchboxes have a portion^✝^ of fruit | **□** Less than half of lunchboxes have a portion^✝^ of fruit | **□** Half or more lunchboxes have at least 1 portion^✝^ of fruit | **□** Almost all (or all) lunchboxes have at least 1 portion^✝^ of fruit |
|  | ^✝^  A portion size for children fits in the palm of their hand. See the ‘Eat Better Start Better’ guidelines for more detail. Fruit can be included as part of a composite meal e.g. banana in custard and can include whole fruit juice. | | | |

| 44. | On a typical day, lunchboxes have vegetables provided as follows: | | | |
| --- | --- | --- | --- | --- |
|  | **□** Few (or no) lunchboxes have a portion^✝^ of vegetable | **□** Less than half of lunchboxes have a portion^✝^ of vegetable | **□** Half or more lunchboxes have at least 1 portion^✝^ of vegetable | **□** Almost all (or all) lunchboxes have at least 1 portion^✝^ of vegetable |
|  | ^✝^ Portion sizes for children are in the ‘Eat Better Start Better’ guidelines. Vegetables can be included as part of a composite meal e.g. mushrooms in pasta. | | | |

For the following statements think about the typical food provided in children’s lunchboxes over a period of five days and consider the food groups and range of food. As children don’t always attend five days a week, for some children this may be five days spread out over a longer period.

| 45. | Four or more different types of vegetable (eg. one day cucumber, another day tomato, another day pepper, another day carrot) are provided in lunchboxes over a five day period: | | | |
| --- | --- | --- | --- | --- |
|  | **□** Few (or no) lunchboxes | **□** Less than half of lunchboxes | **□** Half or more lunchboxes | **□** Almost all (or all) lunchboxes |
|  | For this question, potato is not included as a vegetable, but sweet potato is included. Vegetables can be included as part of a composite meal e.g. mushrooms and pasta. | | | |

| 46. | Four or more different types of fruit (eg. One day apple, another day tangerine, another day banana, another day grapes) are provided in lunchboxes over a five day period: | | | |
| --- | --- | --- | --- | --- |
|  | **□** Few (or no) lunchboxes | **□** Less than half of lunchboxes | **□** Half or more lunchboxes | **□** Almost all (or all) lunchboxes |

| 47. | Four or more different types of proteins (one day fish, another day eggs, another day peas, another day poultry) are provided in lunchboxes over a five day period: | | | |
| --- | --- | --- | --- | --- |
|  | **□** Few (or no) lunchboxes | **□** Less than half of lunchboxes | **□** Half or more lunchboxes | **□** Almost all (or all) lunchboxes |
|  | Examples of types of protein: Poultry, fish, red meat, eggs, pulses, meat alternatives, oily fish. Pulses include beans, chickpeas, lentils. Meat-free alternatives include Quorn pieces or Tofu. To note, oily fish should be included weekly, but should **not** be given more than twice per week. Oily fish includes salmon or tinned salmon, sardines or pilchards. It does not include tinned tuna. | | | |

| 48. | At least three different types of starchy foods are provided in lunchboxes over a five day period (eg. on one day bread, another day rice, another day pasta, another day potato): | | | | | | |  |
| --- | --- | --- | --- | --- | --- | --- | --- | --- |
|  | **□** Few (or no) lunchboxes | **□** Less than half of lunchboxes | | **□** Half or more lunchboxes | | **□** Almost all (or all) lunchboxes | |  |
| 49. | The range of dairy foods^^^ or drinks^^^ incorporating low sugar products^✝^provided in lunchboxes over a five day period is typically: | | | | | | | |
|  | **□** 1 or fewer dairy food/drink | | **□** At least 2 dairy food/drinks | | **□** At least 3 dairy food/drinks | | **□** At least 3 dairy food/drinks | |
|  | ^^^A portion of dairy could include:   - 100-150ml of milk (half a cup) - a matchbox size (20-30g) of cheese - small pot (60g) or half a large individual pot (half of 125-140g) of yoghurt/fromage frais - 1-2 tablespoons of yoghurt from a multi-serving pot - 3-4 tablespoons of a milk-based pudding (custard, rice pudding or semolina)   ^✝^A lower sugar content would be, e.g. for yogurt, no higher than 15g sugar per 100g. | | | | | | | |

For the following statements think about the typical food provided in children’s lunchboxes in a typical day and consider the food groups and range of food.

| 50. | Foods or drinks high in saturated fat*, sugar* and salt^✝^ are provided in lunchboxes on a typical day: | | | |
| --- | --- | --- | --- | --- |
|  | **□** Almost all (or all) lunchboxes contain a food or drink high in saturated fat, sugar or salt | **□** Half or more lunchboxes contain a food or drink high in saturated fat, sugar or salt | **□** Less than half of the lunchboxes contain a food or drink high in saturated fat, sugar or salt | **□** Few (or no) lunchboxes contain a food or drink high in saturated fat, sugar or salt |
|  | * Examples of high-sugar or high-fat snacks include biscuits, cakes, doughnuts, American style muffins, chocolate, sweets or confectionary  ^✝^Such as crisps, tortilla chips, pretzels, salted popcorn  Sugar-sweetened drinks such as orange squash, lemonade | | | |

| 51. | For lunchboxes that include fruit juice as a drink, on a typical day they are provided as follows: | | | |
| --- | --- | --- | --- | --- |
|  | **□** Almost all (or all) lunchboxes contain fruit juice without dilution | **□** Half or more lunchboxes contain fruit juice without dilution | **□** Less than half of the lunchboxes contain fruit juice without dilution | **□** Few (or no) lunchboxes contain fruit juice without dilution |

| 52. | Sugar-sweetened drinks (for example orange squash, lemonade) in lunchboxes on a typical day are provided as follows: | | | |
| --- | --- | --- | --- | --- |
|  | **□** Almost all (or all) lunchboxes contain a sugar-sweetened drink | **□** Half or more lunchboxes contain a sugar-sweetened drink | **□** Less than half of the lunchboxes contain a sugar-sweetened drink | **□** Few (or no) lunchboxes contain a sugar-sweetened drink |

| 53. | Water to drink is provided in lunchboxes on a typical day (or provided in cups by the nursery) in: | | | |
| --- | --- | --- | --- | --- |
|  | **□** Few (or no) lunchboxes | **□** Less than half of the lunchboxes | **□** Half or more lunchboxes | **□** Almost all (or all) lunchboxes |

**Education and Professional Development (additions for lunchboxes)**

| 54. | Professional development for current staff on child nutrition has included the following topics: | | | |
| --- | --- | --- | --- | --- |
|  | *Tick all that apply:*   - Food and drink recommendations for children - Serving sizes for children - Importance of variety in the children’s diet - Lunchbox variety, content and monitoring quality of content - Creating healthy mealtime environments* - Using positive feeding practices to support children to eat well^✝^ - Supporting staff to communicate with families about child nutrition - Your nursery’s policies on child nutrition | | | |
|  | **□** 0 ticks | **□** 1-3 ticks | **□** 4-6 ticks | **□** 7-8 ticks |
|  | *****In a healthy mealtime environment, children can choose what to eat from the foods offered, television and videos are turned off, and staff sit with children and enthusiastically role model eating healthy foods.  ^✝^Positive feeding practices include praising children for trying new foods, asking children about hunger/fullness before taking their plates away or serving seconds, and avoiding the use of food to calm children or to encourage appropriate behaviour. | | | |

| 55. | Information or support for families on child nutrition includes the following topics: | | | |
| --- | --- | --- | --- | --- |
|  | *Tick all that apply:*   - Food and beverage recommendations for children - Serving sizes for children - Importance of variety in the children’s diet - Creating healthy mealtime environments - Using positive feeding practices - Your nursery’s policies on child nutrition - Lunchbox variety, content and monitoring quality of content | | | |
|  | **□** 0 ticks | **□** 1-2 ticks | **□** 3-4 ticks | **□** 5-7 ticks |

**Policy (additions for lunchboxes)**

| 56. | Our written policy* on child nutrition includes the following topics: | | | |
| --- | --- | --- | --- | --- |
|  | *Tick all that apply:*   - Foods provided to children - Beverages provided to children - Creating healthy mealtime environments - Teaching practices to encourage healthy eating - Not offering food to calm children or encourage appropriate behaviour - Planned and informal nutrition education for children - Professional development on child nutrition - Education for families on child nutrition including lunchboxes - Guidelines for food offered for seasonal/religious celebrations, birthdays etc - Provision of special diets - Lunchbox content, portion size and variety - Monitoring lunch box content and quality and giving constructive feedback to parents | | | |
|  | **□** No written policy or policy does not include these topics | **□** 1-6 ticks | **□** 7-9 ticks | **□** 10-12ticks |
|  | *****A written policy can include any written guidance about your nursery’s operations or expectations for staff, children, and families. Policies can be included in parent handbooks, staff manuals, and other documents. Ideally staff, parents and children are involved in writing the policy. | | | |

**Feeding Environment and Practices for lunchbox consumed meals**

| 57. | Television or videos or screens are on during meal times: | | | |
| --- | --- | --- | --- | --- |
|  | **□** Always or almost always | **□** Once a week | **□** Once a fortnight | **□** Rarely or never |

| 58. | Staff eat or drink unhealthy foods or beverages in front of children: | | | |
| --- | --- | --- | --- | --- |
|  | **□** Always or almost always | **□** Once a week | **□** Once a fortnight | **□** Rarely or never |

| 59. | Staff enthusiastically role model* eating healthy foods: | | | |
| --- | --- | --- | --- | --- |
|  | **□** Rarely or never | **□** Once a fortnight | **□** Once a week | **□** Always or almost always |
|  | *Enthusiastic role modelling is when staff eat healthy foods in front of children and show how much they enjoy them. For example, a staff member might say, “Mmmm, these peas taste yummy!” | | | |

| 60. | Staff praise children for trying new or less-preferred foods in their lunchbox: | | | |
| --- | --- | --- | --- | --- |
|  | **□** Rarely or never | **□** Once a fortnight | **□** Once a week | **□** Always or almost always |

| 61. | Children are required to finish everything in their lunchbox: | | | |
| --- | --- | --- | --- | --- |
|  | **□** Always or almost always | **□** Once a week | **□** Once a fortnight | **□** Rarely or never |

| 62. | Children eat their lunchboxes: | | | |
| --- | --- | --- | --- | --- |
|  | **□** In their usual room with no change | **□** In their usual room and some difference to playtime* is *sometimes* used | **□** In their usual room, but meal times are *always* clearly different from playtime* | **□** In a separate room^✝^ or separate area^^^ to where they play |
|  | *To make mealtimes different to learning/play times, a song could be used to mark the change to eating time, toys and equipment could be tidied away and cloths could be put on tables  ^✝^The separate room could be a side room or a dining room  ^^^ This would be a separate area within the room that is used for mealtimes only | | | |

Physical Activity and Play

For this self-assessment, **physical activity** is any movement of the body that increases heart rate and breathing above what it would be if a child was sitting or resting.

Time Provided

| 63. | The amount of time provided for 2-4 year old children who can walk unaided, for indoor and outdoor physical activity* each day is: | | | |
| --- | --- | --- | --- | --- |
|  | **□** Less than 1 hour | **□** 1-2 hours | **□** 2-3 hours | **□** 3 hours or more |
|  | *This physical activity can includes light activity such as walking, rolling, playing, active play and more energetic activity such as running, skipping, jumping and dancing. | | | |

| 64. | The amount of adult-led* physical activity our nursery provides for 2-4 year old children each day is: | | | |
| --- | --- | --- | --- | --- |
|  | **□** Less than 30 minutes | **□** 30-44 minutes | **□** 45-59 minutes | **□** 1 hour or more |
|  | *Adult-led activities and lessons can be led by staff or outside agencies. Examples include dancing, music and movement, motor development lessons, physically active games, gymnastics, active storytelling and forest school activities. The total amount of adult-led activity time may include multiple short activities added up over the course of the day. | | | |

| 65. | Outside of nap and meal times, the longest that children are asked to remain seated at any one time* is: | | | |
| --- | --- | --- | --- | --- |
|  | **□** 30 minutes or more | **□** 20-29 minutes | **□** 15-19 minutes | **□** Less than 15 minutes |
|  | *This does not include free play where a child may choose to sit still e.g. to do a painting.  An example of a time when a child is asked to remain seated would be circle time or story time. | | | |

Indoor Play Environment

| 66. | Our nursery has the following portable play equipment* available and in good condition for children to use **indoors**: | | | |
| --- | --- | --- | --- | --- |
|  | Tick all that apply:   - Jumping toys: skipping ropes, space hoppers - Push-pull toys: wheelbarrows, trolleys, walkers, big trucks - Twirling toys: ribbons, scarves, batons, hula hoops, parachute - Throwing, catching and batting toys: balls, pom poms, bean bags, noodles, rackets - Balance toys: balance beams, stepping stones - Crawling or tumbling equipment: mats, portable tunnels | | | |
|  | **□** 0 ticks | **□** 1-2 ticks | **□** 3-4 ticks | **□** 5-6 ticks |
|  | *****Portable play equipment includes any toys that children can carry, throw, push, pull, etc. to help them build gross motor skills. This does not include equipment fixed to the floor or the walls, but does include fabric tunnels, mats and other larger items that staff can easily move around. Portable play equipment can be homemade or shop bought. | | | |

| 67. | Staff offer portable play equipment to children during indoor free play time: | | | |
| --- | --- | --- | --- | --- |
|  | **□** Sometimes or never | **□** Often | **□** At least a few items are always available to encourage physical activity | **□** Items are always available & children can move the equipment freely between different areas of the room |

Staff Practices

| 68. | To manage challenging behaviour, staff take away time for physical activity or remove children from physically active playtime for longer than 5 minutes: | | | |
| --- | --- | --- | --- | --- |
|  | **□** Always | **□** Often | **□** Sometimes | **□** Never |

| 69. | Staff incorporate physical activity into daily routines, transitions between activities, and other planned activities:* | | | |
| --- | --- | --- | --- | --- |
|  | **□** Rarely or never | **□** Sometimes | **□** Often | **□** Each time they see an opportunity |
|  | *Physical activity during routines, transitions and planned activities can include playing Simon Says or other movement games while children wait in a queue or transition between activities, or using movement during circle time or story time. | | | |

| 70. | Staff role-model good physical activity habits* | | | |
| --- | --- | --- | --- | --- |
|  | **□** Rarely or never | **□** Sometimes | **□** Often | **□** Each time they see an opportunity |
|  | *Good physical activity habits include joining in with both child led and adult led active play and structured physical activity sessions with enthusiasm and wearing appropriate clothing for engaging in physical activities. | | | |

| 71. | We encourage families and staff to actively commute* to our nursery in the following ways: | | | |
| --- | --- | --- | --- | --- |
|  | Tick all that apply:   - Storage facilities are provided for pushchairs to be left safely during the day - Storage facilities are provided for children’s scooters and bikes to be left safely during the day - Bike storage, changing facilities and lockers/storage are available for staff who have actively commuted and wish to change and store their clothes - Posters, leaflets, website and/or newsletters are used to promote active commuting to our families - Children are taught about road safety, traffic awareness and how to cross the road | | | |
|  | **□** 0-2 ticks | **□** 3 ticks | **□** 4 ticks | **□** 5 ticks |
|  | *****Active commuting includes any form of transport that requires the person to be physically active and can include walking, cycling, scooting | | | |

Education and Professional Development

| 72. | Children participate in planned activities focused on building gross motor skills:* | | | |
| --- | --- | --- | --- | --- |
|  | **□** Less than once per week | **□** Once per week | **□** 2-3 times per week or more | **□** Daily |
|  | *Gross motor skills are physical abilities and large muscle control that children develop as they grow. Activities to build gross motor skills may focus on children skipping, jumping, throwing, catching, kicking, balancing, stretching, or other specific skills. | | | |

| 73. | Staff talk with children informally & formally about the importance of physical activity: | | | |
| --- | --- | --- | --- | --- |
|  | **□** Rarely or never | **□** Sometimes | **□** Often | **□** Each time they see an opportunity |

| 74. | Staff receive professional development*on children’s physical activity: | | | |
| --- | --- | --- | --- | --- |
|  | **□** Never | **□** Less than once per year | **□** Once per year | **□** 2 times per year or more |
|  | *For this question, professional development on children’s physical activity does not include training on playground safety. Professional development can include in-person or online training for contact hours or continuing professional development credits. It can also include information presented at staff meetings. Training should be from a reputable source (e.g. NHS, Local Authority or National Organisation). | | | |

| 75. | Professional development for current staff on children’s physical activity has included the following topics: | | | |
| --- | --- | --- | --- | --- |
|  | Tick all that apply:   - Recommended amounts of daily physical activity for young children - Encouraging children’s physical activity - Limiting long periods of seated time for children - Children’s motor skill development - Communicating with families about encouraging children’s physical activity - Your nursery’s policies on physical activity and active travel | | | |
|  | **□** 0 ticks | **□** 1-2 ticks | **□** 3-4 ticks | **□** 5-6 ticks |

| 76. | Information or education for families on children’s physical activity includes the following topics: | | | |
| --- | --- | --- | --- | --- |
|  | Tick all that apply:   - Recommended amounts of daily physical activity for young children - Encouraging children’s physical activity - Limiting long periods of seated time for children - Children’s motor skill development - Your nursery’s policies on physical activity | | | |
|  | **□** 0 ticks | **□** 1 tick | **□** 2-3 ticks | **□** 4-5 ticks |
|  | *Information or education can be offered through in-person educational sessions, brochures, leaflets, tip sheets, or your nursery’s newsletter, website, bulletin board or Facebook page. | | | |

Policy

| 77. | Our written policy* on physical activity includes the following topics: | | | |
| --- | --- | --- | --- | --- |
|  | Tick all that apply:   - Amount of time provided each day for indoor and outdoor physical activity - Limiting long periods of seated time for children - Shoes and clothes that allow children and staff to actively participate in physical activity - Staff practices that encourage physical activity - Not taking away physical activity time or removing children from long periods of physically active playtime in order to manage challenging behaviours - Planned and informal physical activity education - Professional development on children’s physical activity - Education for families on children’s physical activity - Active travel | | | |
|  | **□** No written policy or policy does not include these topics | **□** 1-3 ticks | **□** 4-6 ticks | **□** 7-9 ticks |
|  | *A written policy can include any written guidelines about your nursery’s operations or expectations for staff, children and families. Policies can be included in parent handbooks, staff manuals, and other documents. | | | |

 Outdoor Play and Learning

For this self-assessment, **outdoor play and learning** includes all activities done outdoors. The questions cover a range of activities, some focused on physical activity and some focused on other learning activities.

Outdoor Play

| 78. | Outdoor playtime* is provided to preschool children: | | | |
| --- | --- | --- | --- | --- |
|  | **□** Once per day | **□** 2 times per day | **□** 3 times per day | **□** 4 times per day or more / ‘free flow’ play |
|  | * Outdoor playtime includes any time that children are outdoors playing and learning. Children may be very physically active or do less energetic activities during this time. | | | |

| 79. | The amount of outdoor playtime provided to children each day is: | | | |
| --- | --- | --- | --- | --- |
|  | **□** Less than 1 hour | **□** 1-2 hours | **□** 2-3 hours | **□** More than 3 hours |

| 80. | When it is very wet outside: | | | |
| --- | --- | --- | --- | --- |
|  | **□** Children stay indoors | **□** Only children who have their own waterproof clothing can go outside | **□** We have some waterproof clothing available so some children can go out | **□** All children have waterproof clothing* and go outside regardless of rain |
|  | * Either provided by the nursery, or brought in from home | | | |

| 81. | Our nursery does the following types of activities with children outdoors: | | | |
| --- | --- | --- | --- | --- |
|  | Tick all that apply:   - Free play: Playtime that can be more or less energetic, depending on what activities children decide to do. - Structured learning opportunities: Planned lessons and activities including circle time, art projects, and reading time. - Seasonal outdoor activities: Activities that are unique to the season or the weather, including gardening, water play, collecting fallen leaves, and playing in the snow. - Walking trips: Activities, like nature walks, ‘welly walks’ and neighbourhood tours, which let children explore the outdoors close to your nursery, but beyond their usual play space. - Outdoor field trips: Opportunities for children to take part in outdoor activities in the local area. Destinations can include local parks, woodland, farms, gardens, zoos or nature reserves. | | | |
|  | **□** 0 ticks | **□** 1 tick | **□** 2-3 ticks | **□** 4-5 ticks |

Outdoor Physical Environment

| 82. | The area we access for outdoor area (at the nursery or another outdoor area) for games, activities & events is: | | | |
| --- | --- | --- | --- | --- |
|  | **□** Large enough for *some* children to run around safely | **□** Large enough for *most* children to run around safely | **□** Large enough for *all* children to run around safely*^✝^ | **□** Large enough for *all* children to run around safely*^✝^ and we also regularly access a range of other outdoor areas to provide variety |
|  | * This refers to all children who regularly use the outdoor area together, not necessarily all of the children in the nursery. For large nurseries, this response refers to a space large enough for at least 25 children to run around safely.  ^✝^This option also applies if the nursery has a small outside space, but supplement it by daily trips to a local park/garden to ensure all children have room to run around safely. | | | |

| 83. | The outdoor space* which the children use includes: | | | |
| --- | --- | --- | --- | --- |
|  | **□** 1-2 play opportunities^✝^ | **□** 3-4 play opportunities ^✝^ | **□** 5-6 or more play opportunities^✝^ | **□** 7 or more play opportunities^✝^ *which change at least monthly* |
|  | * The outdoor space can be the nursery’s own garden, or for nurseries with no outdoor space, it can be a local park or garden if used regularly  ^✝^Each play opportunity offers different play opportunities. An area might include a swing set, sand pit, climbing frame, pathway, garden, Wendy house or tent, paddling pool, easel or outdoor musical instruments like pots and pans, mud kitchen or Forest School area. A play area does not need to be permanent; it can be created by bringing equipment outside. | | | |

| 84. | Describe your nursery’s space to grow herbs, fruit and/or vegetables* | | | |
| --- | --- | --- | --- | --- |
|  | **□** There is no garden for herbs, fruit or vegetables | **□** It grows only herbs | **□** It grows some fruit and/or vegetables for children to taste | **□** It grows enough fruit and vegetables to provide children meals or snacks during 1 or more seasons |
|  | * A space to grow herbs, fruit and/or vegetables can be planted in the ground or in containers like window boxes, grow-bags or pots, or fruit trees planted in the outdoor play space. | | | |

| 85. | In your nursery, the path* for wheeled toys is: | | | |
| --- | --- | --- | --- | --- |
|  | **□** No path | **□** Unpaved and any width | **□** Paved and less than 1.5m wide | **□** Paved and 1.5m wide or wider |
|  | *This can include a painted or drawn-on path | | | |

| 86. | Describe the shape of your path* for ride-on or push-along toys: | | | |
| --- | --- | --- | --- | --- |
|  | **□** No path | **□** Straight | **□** Circular | **□** Figure of 8-shaped or with multiple loops* |
|  | *A Figure of 8-shaped path allows children to ride around multiple loops, not just one large circle. The path can include a painted or drawn-on path. | | | |

| 87. | Describe how the path for wheeled toys connects to different parts of the outdoor play space: | | | |
| --- | --- | --- | --- | --- |
|  | Tick all that apply:   - Connects to building entrances - Connects the building to play areas - Connects different play opportunities to each other | | | |
|  | **□** No path | **□** 1 tick | **□** 2 ticks | **□** 3 ticks |

| 88. | Our nursery has the following portable play equipment* available and in good condition for children to use **outdoors:** | | | |
| --- | --- | --- | --- | --- |
|  | Tick all that apply:   - Jumping toys: skipping ropes, space hoppers - Push-pull toys: wheelbarrows, trolleys, walkers, big trucks - Ride-on toys: tricycles, scooters, balance bikes - Twirling toys: ribbons, scarves, batons, hula hoops, parachute - Throwing, catching and batting toys: balls, pom poms, bean bags, noodles, rackets - Balance toys: balance beams, stepping stones - Crawling or tumbling equipment: mats, portable tunnels - Other “loose parts”: sticks, shovels, buckets | | | |
|  | **□** 0 types | **□** 1-3 types | **□** 4-6 types | **□** 7-8 types |
|  | *****Portable play equipment includes any toys or natural resources that children can carry, throw, push, pull, or kick, as well as “loose parts” that help children explore and learn about the natural world. Portable play equipment does not include equipment fixed to the ground like climbing frames, but does include fabric tunnels, mats and other larger items that staff can easily move around. Portable play equipment can be homemade or shop bought. | | | |

| 89. | Portable play equipment is available to children during outdoor active playtime: | | | |
| --- | --- | --- | --- | --- |
|  | **□** Rarely or never | **□** Sometimes | **□** Often | **□** Always |

| 90. | The amount of portable play equipment available to children during outdoor active playtime is: | | | |
| --- | --- | --- | --- | --- |
|  | **□** Very limited | **□** Limited | **□** Somewhat limited | **□** Not limited – there is always something available for each child to play with |

Education & Professional Development

| 91. | Practitioners and other staff receive professional development* on **outdoor** play and learning: | | | |
| --- | --- | --- | --- | --- |
|  | **□** Never | **□** Less than once per year | **□** Once per year | **□** Two times per year or more |
|  | * Professional development can include taking in-person or online training for contact hours or continuing education credits. It can also include information presented at staff meetings. Training should be from a reputable source (e.g. NHS, Local Authority or National Organisation). | | | |

| 92. | Professional development for current staff on outdoor play and learning has included the following topics: | | | |
| --- | --- | --- | --- | --- |
|  | Tick all that apply:   - Recommended amounts of outdoor playtime for young children - Using the outdoor play space to encourage children’s physically active play - Communicating with families about outdoor play and learning - Our nursery’s policies on outdoor play and learning | | | |
|  | **□** None | **□** 1 tick | **□** 2-3 ticks | **□** 4 ticks |

| 93. | Information or education for families on outdoor play and learning includes the following topics: | | | |
| --- | --- | --- | --- | --- |
|  | Tick all that apply:   - Recommended amounts of outdoor playtime for young children - Using outdoor play space to encourage children’s physically active play - Our nursery’s policies on outdoor play and learning | | | |
|  | **□** None | **□** 1 tick | **□** 2 ticks | **□** 3 ticks |
|  | *Information or education can be offered through in-person education sessions, brochures, tip sheets, or your nursery’s newsletter, website or bulletin board. | | | |

Please continue to question 94 on the next page

Policy

| 94. | Our written policy* on outdoor play and learning includes the following topics: | | | |
| --- | --- | --- | --- | --- |
|  | Tick all that apply:   - Amount of outdoor playtime provided each day - Ensuring adequate total playtime on bad^✝^ weather days - Shoes and clothes that allow children and staff to play outdoors in all seasons - Safe sun exposure for children, staff, and staff - Not taking away outdoor playtime in order to manage challenging behaviours - Professional development on outdoor play and learning - Education for families on outdoor play and learning | | | |
|  | **□** No written policy or policy does not include these topics | **□** 1-2 ticks | **□** 3-5 ticks | **□** 6-7 ticks |
|  | *****A written policy includes any written guidelines about your nursery’s operations or expectations for staff, children, and families. Policies can be included in parent handbooks, staff manuals, and other documents. Your policy on outdoor play and learning may be found in the physical activity policy or health and safety policy or may be a separate policy entirely. | | | |
|  | ^✝^Bad weather days include very high and very low temperatures, high winds, heavy rain or storms, snow, and any other factors that make the outdoors unsafe for children | | | |

 Screen Time

For this self-assessment, **screen time** includes any time spent watching TV programmes, DVDs or playing games (including active video games) on a screen. Screens can include televisions; desktop, laptop, or tablet computers; or smart phones. Screen time **does not** include staffs using e-books or tablet computers to read children stories or using Smart/White Boards for interactive instruction.

Availability & Staff Practices

| 95. | Televisions are located: | | | |
| --- | --- | --- | --- | --- |
|  | **□** In every room | **□** In some rooms | **□** Stored outside rooms but regularly available to children | **□** No televisions; or, televisions stored outside of rooms and not regularly available to children |

| 96. | For children age 2 -4 years, the amount of screen time* allowed in our nursery per child, per week is: | | | |
| --- | --- | --- | --- | --- |
|  | **□** 90 minutes or more | **□** 60-89 minutes | **□** 30-59 minutes | **□** Less than 30 minutes or no screen time allowed |
|  | *Screen time includes television/DVD viewing and use of computers, laptops, e-books, smart phones or tablet computers. Screen time **does not** include staff using e-books or tablet computers to read children stories, using Smart/White Boards for interactive instruction. | | | |

| 97. | When television or DVDs are shown to children, the programmes shown are educational and advertisement free*: | | | |
| --- | --- | --- | --- | --- |
|  | **□** Rarely or never | **□** Sometimes | **□** Often | **□** Always |
|  | *Education and advertisement free programmes and DVDs are developmentally appropriate, support children’s learning goals and do not contain any advertising. | | | |

| 98. | When screen time is offered, children are given the opportunity to do an alternative activity: | | | |
| --- | --- | --- | --- | --- |
|  | **□** Rarely or never | **□** Sometimes | **□** Often | **□** Always |

Education & Professional Development

| 99. | Professional development for current staff on screen time has included the following topics: | | | |
| --- | --- | --- | --- | --- |
|  | Tick all that apply:   - Recommended amounts of screen time for young children - Appropriate types of programmes for young children - Appropriate supervision and use of screen time in the classroom - Communicating with families about healthy screen time habits - Our nursery’s policies on screen time | | | |
|  | **□** 0 ticks | **□** 1-2 ticks | **□** 3-4 ticks | **□** 5 ticks |

| 100. | Information or education* for families on screen time includes the following topics: | | | |
| --- | --- | --- | --- | --- |
|  | Tick all that apply:   - Recommended amounts of screen time for young children - Appropriate types of programmes for young children - Appropriate supervision and use of screen time by parents/carers - Our nursery’s policies on outdoor play and learning | | | |
|  | **□** 0 ticks | **□** 1-2 tick | **□** 3 ticks | **□** 4 ticks |
|  | *Information or education can be offered through in-person education sessions, brochures, tip sheets, or your nursery’s newsletter, website or bulletin board. | | | |

Policy

| 101. | Our written policy* on screen time includes the following topics: | | | |
| --- | --- | --- | --- | --- |
|  | Tick all that apply:   - Amount of screen time allowed - Types of programming allowed - Appropriate supervision and use of screen time in classrooms - Not using screen time as a reward or to manage challenging behaviours - Professional development on screen time - Education for families on screen time | | | |
|  | **□** No written policy or policy does not include these topics | **□** 1-2 ticks | **□** 3-4 ticks | **□** 5-6 ticks |
|  | *****A written policy includes any written guidelines about your nursery’s operations or expectations for staff, children, and families. Policies can be included in parent handbooks, staff manuals, and other documents. Your policy on screen time may be found in the physical activity policy or health and safety policy or may be a separate policy entirely. | | | |

## **References**

1. Goddard, J., *Early years education: Trends, issues and the impact of Covid-19*. 2023.

2. Martin C., H.C., Stevenson S. and Pollok M, *The Contribution of EU Workers in the Social Care Workforce in Scotland 2022*. 2022.

3. Puyau, M.R., et al., *Validation and calibration of physical activity monitors in children.* *Obes Res, 2002. 10(3): p. 150-7.*

4. Pate, R.R., et al., An Intervention to Increase Physical Activity in Children: A Randomized Controlled Trial With 4-Year-Olds in Preschools. Am J Prev Med, 2016. 51(1): p. 12-22.

5. K, W.P.a.N., *The ‘cost of living crisis’.*  Journal of Public Health, 2022. **44**(3): p. 475-476.

6. Education., D.f., *Childcare and early years survey of parents*. 2024.

7. Government, S., *Policy: Early education and care (Early learning and childcare expansion programme*. 2024.

8. Education, D.f., *Childcare and early years providers survey*. 2024.

9. Dias, K., et al., *Acceptability, internal consistency and test-retest reliability of scales to assess parental and nursery staff's self-efficacy, motivation and knowledge in relation to pre-school children's nutrition, oral health and physical activity.* Public Health Nutr, 2019. **22**(6): p. 967-975.
